# MEIOK21: a new component of meiotic recombination bridges required for spermatogenesis

**DOI:** 10.1093/nar/gkaa406

**Published:** 2020-05-28

**Authors:** Yongliang Shang, Tao Huang, Hongbin Liu, Yanlei Liu, Heng Liang, Xiaoxia Yu, Mengjing Li, Binyuan Zhai, Xiao Yang, Yudong Wei, Guoqiang Wang, Zijiang Chen, Shunxin Wang, Liangran Zhang

**Affiliations:** Center for Reproductive Medicine, School of Medicine, Cheeloo College of Medicine, Shandong University, National Research Center for Assisted Reproductive Technology and Reproductive Genetics, Key Laboratory of Reproductive Endocrinology of Ministry of Education, Shandong Provincial Clinical Medicine Research Center for Reproductive Health, Jinan, Shandong 250012, China; Center for Reproductive Medicine, School of Medicine, Cheeloo College of Medicine, Shandong University, National Research Center for Assisted Reproductive Technology and Reproductive Genetics, Key Laboratory of Reproductive Endocrinology of Ministry of Education, Shandong Provincial Clinical Medicine Research Center for Reproductive Health, Jinan, Shandong 250012, China; Center for Reproductive Medicine, School of Medicine, Cheeloo College of Medicine, Shandong University, National Research Center for Assisted Reproductive Technology and Reproductive Genetics, Key Laboratory of Reproductive Endocrinology of Ministry of Education, Shandong Provincial Clinical Medicine Research Center for Reproductive Health, Jinan, Shandong 250012, China; Center for Reproductive Medicine, School of Medicine, Cheeloo College of Medicine, Shandong University, National Research Center for Assisted Reproductive Technology and Reproductive Genetics, Key Laboratory of Reproductive Endocrinology of Ministry of Education, Shandong Provincial Clinical Medicine Research Center for Reproductive Health, Jinan, Shandong 250012, China; Center for Reproductive Medicine, School of Medicine, Cheeloo College of Medicine, Shandong University, National Research Center for Assisted Reproductive Technology and Reproductive Genetics, Key Laboratory of Reproductive Endocrinology of Ministry of Education, Shandong Provincial Clinical Medicine Research Center for Reproductive Health, Jinan, Shandong 250012, China; Center for Reproductive Medicine, School of Medicine, Cheeloo College of Medicine, Shandong University, National Research Center for Assisted Reproductive Technology and Reproductive Genetics, Key Laboratory of Reproductive Endocrinology of Ministry of Education, Shandong Provincial Clinical Medicine Research Center for Reproductive Health, Jinan, Shandong 250012, China; Center for Reproductive Medicine, School of Medicine, Cheeloo College of Medicine, Shandong University, National Research Center for Assisted Reproductive Technology and Reproductive Genetics, Key Laboratory of Reproductive Endocrinology of Ministry of Education, Shandong Provincial Clinical Medicine Research Center for Reproductive Health, Jinan, Shandong 250012, China; Center for Reproductive Medicine, School of Medicine, Cheeloo College of Medicine, Shandong University, National Research Center for Assisted Reproductive Technology and Reproductive Genetics, Key Laboratory of Reproductive Endocrinology of Ministry of Education, Shandong Provincial Clinical Medicine Research Center for Reproductive Health, Jinan, Shandong 250012, China; Advanced Medical Research Institute, Shandong University, Jinan, Shandong 250014, China; Center for Reproductive Medicine, School of Medicine, Cheeloo College of Medicine, Shandong University, National Research Center for Assisted Reproductive Technology and Reproductive Genetics, Key Laboratory of Reproductive Endocrinology of Ministry of Education, Shandong Provincial Clinical Medicine Research Center for Reproductive Health, Jinan, Shandong 250012, China; Center for Reproductive Medicine, School of Medicine, Cheeloo College of Medicine, Shandong University, National Research Center for Assisted Reproductive Technology and Reproductive Genetics, Key Laboratory of Reproductive Endocrinology of Ministry of Education, Shandong Provincial Clinical Medicine Research Center for Reproductive Health, Jinan, Shandong 250012, China; Center for Reproductive Medicine, School of Medicine, Cheeloo College of Medicine, Shandong University, National Research Center for Assisted Reproductive Technology and Reproductive Genetics, Key Laboratory of Reproductive Endocrinology of Ministry of Education, Shandong Provincial Clinical Medicine Research Center for Reproductive Health, Jinan, Shandong 250012, China; Center for Reproductive Medicine, School of Medicine, Cheeloo College of Medicine, Shandong University, National Research Center for Assisted Reproductive Technology and Reproductive Genetics, Key Laboratory of Reproductive Endocrinology of Ministry of Education, Shandong Provincial Clinical Medicine Research Center for Reproductive Health, Jinan, Shandong 250012, China; Center for Reproductive Medicine, School of Medicine, Cheeloo College of Medicine, Shandong University, National Research Center for Assisted Reproductive Technology and Reproductive Genetics, Key Laboratory of Reproductive Endocrinology of Ministry of Education, Shandong Provincial Clinical Medicine Research Center for Reproductive Health, Jinan, Shandong 250012, China; Center for Reproductive Medicine, School of Medicine, Cheeloo College of Medicine, Shandong University, National Research Center for Assisted Reproductive Technology and Reproductive Genetics, Key Laboratory of Reproductive Endocrinology of Ministry of Education, Shandong Provincial Clinical Medicine Research Center for Reproductive Health, Jinan, Shandong 250012, China; Advanced Medical Research Institute, Shandong University, Jinan, Shandong 250014, China; State Key Laboratory of Microbial Technology, Shandong University, Qingdao 266237, China

## Abstract

Repair of DNA double-strand breaks (DSBs) with homologous chromosomes is a hallmark of meiosis that is mediated by recombination ‘bridges’ between homolog axes. This process requires cooperation of DMC1 and RAD51 to promote homology search and strand exchange. The mechanism(s) regulating DMC1/RAD51-ssDNA nucleoprotein filament and the components of ‘bridges’ remain to be investigated. Here we show that MEIOK21 is a newly identified component of meiotic recombination bridges and is required for efficient formation of DMC1/RAD51 foci. MEIOK21 dynamically localizes on chromosomes from on-axis foci to ‘hanging foci’, then to ‘bridges’, and finally to ‘fused foci’ between homolog axes. Its chromosome localization depends on DSBs. Knockout of *Meiok21* decreases the numbers of HSF2BP and DMC1/RAD51 foci, disrupting DSB repair, synapsis and crossover recombination and finally causing male infertility. Therefore, MEIOK21 is a novel recombination factor and probably mediates DMC1/RAD51 recruitment to ssDNA or their stability on chromosomes through physical interaction with HSF2BP.

## INTRODUCTION

Meiosis is a fundamental event of sexual reproduction. Failures in meiosis are the leading cause of infertility and birth defects (1–3). During meiosis, progenitor cells undergo a single round of DNA replication, followed by two successive rounds of cell division to generate haploid gametes. DNA crossover recombination (CO) is a crucial event of meiosis that not only promotes the exchange of genetic information between parents but also establishes the physical connections between homologous chromosomes (homologs), required for proper chromosome segregation (4).

Meiotic recombination is initiated by SPO11 complex-mediated programmed DNA double-strand breaks (DSBs) (5–11). After SPO11 complex and its binding oligos are removed by MRE11, the DSB ends are further resected primarily by the MRE11-RAD50-NBS1 exonuclease complex to generate single strand 3′ overhangs (9,12). This single stranded DNA (ssDNA) is protected by replication protein A (RPA), and then RecA-like proteins DMC1 and RAD51 are recruited to promote the formation of displacement loops (D-loops) (13,14). A small fraction of D-loops is selected to generate single-end invasions (SEIs). However, the majority of D-loops develop into non-crossovers (NCOs) (12,15). SEIs are CO specific recombination intermediates, which will develop into double-Holliday junctions (dHJs) (15). Normally, dHJs are specifically resolved to COs by the MLH1-MLH3 complex (12,15). The process of CO recombination is tightly regulated by a set of factors, especially the well-known ‘ZMM’ proteins, which include Zip1-4, Msh4-5, Mer3 and Spo16 in budding yeast (12,15–17). Absence of or aberrantly located COs tend to result in chromosome mis-segregation and thus aneuploidy (1,2,12,15,18).

Recombinase DMC1 and its accessory factor RAD51 mediate the central step of meiotic DSB repair by catalyzing the nucleoprotein filament to search and invade its homolog partner to form D-loops (13,19). The mechanism(s) regulating DMC1/RAD51-ssDNA filaments remain(s) to be elucidated. Studies have identified several factors involved in this process, including HOP2-MND1, HSF2BP, TEX15, ATR, BRCA1, BRCA2, MEIOB and SWS1-SWSAP1 (20–29). However, how these factors collaborate and recruit DMC1/RAD51 to recombination sites is unknown. One study has shown that without HSF2BP, both DMC1/RAD51 and BRCA2 foci are almost abolished. The authors proposed that HSF2BP interacts with BRCA2, and thus recruits BRCA2-DMC1/RAD51 to DSB sites (28,30). However, *Brca2* mutants show meiosis failures in both males and females. Paradoxically, *Hsf2bp* mutants show severe meiosis defects only in males but not in females (22,28,30). Therefore, how HSF2BP regulates DMC1/RAD51 foci still remains unclear.

During meiosis, one long-lasting question is how RAD51/DMC1-ssDNA nucleoprotein filaments move recombination factors forward to search and invade its homologous DNA. A recombination ‘bridge’ model has been proposed and further elaborated recently (31,32). In this model, cytologically visualized bridge-like structures have been thought to mediate the movement of meiotic recombination factors (31,32). These bridge-like structures are built between the two axes of homologs in the zygotene stage, consisting of DNA, axis proteins and recombination-related proteins, e.g. Spo76/Pds5, Mer3-Msh4 and the Zip2-4 complex in the fungus *Sordaria* (31). Recombination bridges are found in diverse species from fungi and plants to mammals (31). Currently, however, only a few proteins have been found to be involved in recombination bridges. Therefore, identification of new components of recombination bridges will help us to further understand their formation and clarify the mechanisms of meiotic DSB repair.

In this study, we identified a previously uncharacterized protein, as a novel meiotic recombination factor and a component of recombination bridges, which we termed **meio**sis-specific *4930432****K21****RIK* (*Meiok21*). During meiosis, MEIOK21 is recruited to chromosomes and localizes on recombination sites in a DSB-dependent manner. MEIOK21 first appears on the chromosome axis at leptotene. Along with the progression of meiotic recombination, MEIOK21 is released from the axis to form bridge-like structures linking homolog axes before they are synapsed. Finally, MEIOK21 foci are located between synapsed homolog axes and on the synaptonemal complex (SC). Knockout of *Meiok21* results in male mice infertility. Additional experiments showed that greatly decreased DMC1/RAD51 focus number on chromosomes disrupts DSB repair, synapsis and crossover formation in *Meiok21^−^^/^^−^* mice. Furthermore, MEIOK21 physically interacts with HSF2BP, and in *Meiok21^−^^/^^−^* spermatocytes, the loading of HSF2BP to recombination sites is also sharply reduced. Therefore, our results suggest that through interaction with HSF2BP, MEIOK21 regulates DMC1/RAD51-ssDNA nucleoprotein filaments to promote meiotic homologous recombination.

## MATERIALS AND METHODS

### Animals

*Meiok21* knockout mice were constructed using the CRISPR/Cas9 system. The mouse *4930432K21RIK* gene (GenBank: NM_029045.2; Ensembl: ENSMUSG00000008129) is located on chromosome 8 and contains nine exons with the start codon in the second exon and the stop codon in the last exon. To construct the *Meiok21* knockout mice, exon 3 to exon 6 were deleted. All WT and knockout mice had a C57BL/6 background. The gene knockout founders were genotyped by PCR followed by DNA sequencing analysis. The homozygous mutant mice were generated by intercross of heterozygous mutants.

The *Spo11* knockout mice were kindly provided by Prof. Hongbin Liu from Shandong University, and *Dmc1* knockout mice were kindly provided by Prof. Minghan Tong from the Shanghai Institute of Biochemistry and Cell Biology, CAS.

The use of mice was approved by the Animal Ethics Committee of the School of Medicine, Shandong University. All animal care protocols and experiments in this study were reviewed and approved by the Animal Use Committee of the School of Medicine, Shandong University.

### Genotyping

Mutant mice genotyping was performed by PCR amplification of genomic DNA extracted from mouse tail tips (33). The PCR primers for the *Meiok21* mutant allele were: (Forward) 5′-AAT CTT GGG AGC TGG AGT ATG CTC TG-3′ and (Reverse) 5′-CCT TCA GCC CCT CTA CTA CAC ACT ATG-3′, and the PCR product was 710 bp. PCR primers for the wild-type *Meiok21* allele were: (Forward) 5′- AGG GAG AGA TAG GAA GCC TGT CAG TC-3′ and (Reverse) 5′-CCT TCA GCC CCT CTA CTA CAC ACT ATG-3′, and the PCR product was 469 bp.

### Plasmids and antibodies

Full length *Meiok21* cDNA was cloned from mouse testis cDNA by PCR. The PCR product bearing *Meiok21* and pGBKT7 vector plasmid were digested with EcoRI and XhoI and ligated to produce *Meiok21*-pGBKT7, which was confirmed by sequencing. Full length or truncated *Meiok21* cDNA was subcloned from *Meiok21*-pGBKT7 plasmid by PCR. After digestion by EcoRI and XmaI, they were ligated with pCAG-GFP vector digested by the same restriction enzymes. Full length or truncated *Hsf2bp* cDNA was cloned from mouse testis cDNA by PCR and inserted into PRK vector after digestion by SalI and NotI.

Rabbit and guinea pig anti-MEIOK21 polyclonal antibody was prepared against amino acids 1–230 of mouse MEIOK21 protein by Dai-an Biological Technology Incorporation (Wuhan, China) as previously described (34). Primary antibodies used for immunostaining were as follows: rabbit anti-MEIOK21 (1:200), guinea pig anti-MEIOK21 (1:100, coimmunostaining with RPA2, RAD51, DMC1 or HSF2BP), mouse anti-SYCP3 (1:800; Abcam #ab97672), rabbit anti-SYCP1 (1:800; Abcam #ab15090), rabbit anti-RPA2 (1:500; Abcam #ab76420), rabbit anti-RAD51 (1:100; Thermo Fisher Scientific #PA5-27195), rabbit anti-DMC1 (1:100; Proteintech #13714-1-AP50), mouse anti-phospho-Histone H2A.X (Ser139) (clone JBW301, 1:300; Millipore #05-636), mouse anti-MLH1 (1:100; BD Biosciences #550838), rabbit anti-HSF2BP (1:200; Abcam #ab126252), rabbit anti-BRCA2 (1:200; Abcam #ab123491). Secondary antibodies were: Alexa Fluor 594-conjugated goat anti-mouse (1:500; Proteintech, #CL594-66467), Alexa Fluor 405-conjugated donkey anti-mouse (1:500; Abcam, #175658), Alexa Fluor 488-conjugated goat anti-rabbit (1:500; Abcam, #ab150077), Alexa Fluor 594-conjugated goat anti-rabbit (1:500; Abcam, #150088), Alexa Fluor 488-conjugated goat anti-guinea pig (1:500; Abcam, #150185).

### Fertility test

Fertility was tested in male mice of the indicated genotypes (8–12 weeks, *n* = 5). Each male mouse was caged with two wild-type C57BL/6 females (4–6 weeks), that were checked for vaginal plugs every morning. Once a vaginal plug was identified (day 1 postcoitus), the male was allowed to rest for 2 days, then another female was placed in the cage for another round of mating. The plugged female was separated and singly caged, and the pregnancy was recorded. If a female did not generate any pups by day 22 postcoitus, it was deemed as not pregnant and euthanized to confirm that result. The fertility test lasted for 2 months.

### Yeast two hybrid assay

Yeast two-hybrid assay (Y2H) was performed as described previously (35). Mouse *Meiok21* cDNA was subcloned into pGADT7 vector as prey. Mouse *Rad51*, *Dmc1, Hsf2bp*, *Rpa1*, *Rpa2*, *Rpa3*, *Spata22* and *Meiob* cDNA were separately subcloned into pGBKT7 vector as bait. The bait and prey plasmids were co-transformed into Y2H gold strain and the interaction was tested on SD-Leu-Trp-His plates.

### Immunoprecipitation assay

Testes (∼200 mg) or 2 × 10^7^ transfected HEK-293T cells were treated with lysis buffer (50 mM Tris–HCl, pH 8.0, 120 mM NaCl, 20 mM NaF, 20 mM β-glycerophosphate, 1 mM EDTA, 6 mM EGTA, pH 8.0, 1% NP-40, 1 mM DTT) on ice for 30 min, then centrifuged at 12 000 × *g* for 10 min at 4°C. About 5% of the supernatant was used as input. Half of the remaining supernatant was incubated with ∼2 μg of the corresponding primary antibody and the other half was incubated with ∼2 μg IgG overnight. Then the antibodies were conjugated with protein A/G-magnification beads (MedChemExpress, HY-K0202) for 2 h. After washing, SDS loading buffer was added to the beads and the mixture was boiled at 95°C for 5 min. Samples were separated by SDS-PAGE and immunoblotted with indicated primary and secondary antibodies.

### Histological and surface nuclear spread analyses

Mice testes and cauda epididymis were isolated immediately after euthanasia, fixed in Bouin's solution (Sigma, #HT10132-1L) for 24 h, and then embedded in paraffin. Five-micron sections were prepared and mounted on glass slides. After deparaffinization, slides were stained with hematoxylin and eosin (HE) for histological analysis. TUNEL staining was performed with TUNEL kit (keyGEN BioTECH, #KGA7072) following the manufacturer's instructions. PAS staining was performed with Periodic Acid Schiff (PAS) Stain Kit (Abcam, ab150680). Stages of seminiferous epithelium cycle and spermatid development were determined as previously described (36). For chromosome spread, testicular tubules were pretreated by hypotonic buffer (30 mM Tris, 50 mM sucrose, 17 mM trisodium citrate dihydrate, 5 mM EDTA, 0.5 mM DTT and 0.5 mM phenylmethylsulphonyl fluoride (PMSF), pH 8.2) for 30 min. Short fragments of testicular tubules were suspended in 0.1 M sucrose and dispersed to single cells, then spread to a thin cell layer on glass slides, and treated with 1% (w/v) paraformaldehyde solution containing 0.15% Triton X-100 (37).

### Imaging

Immunostained slides were imaged by confocal microscopy (Andor Dragonfly spinning disc confocal microscope driven by Fusion Software). Histology results were analyzed using an epifluorescence microscope (BX52, Olympus). Super-resolution structured illumination microscopy (SIM) analysis was performed using Acquire SR software on a DeltaVision OMX SR super resolution imaging system (GE Healthcare) equipped with a 60×/1.42 oil objective, and the images were further computationally reconstructed and processed with softWoRx software (GE Healthcare) to generate super resolution optical series sections with two-fold extended resolution in *x*, *y* and *z* axes.

### Reverse transcriptional PCR (RT-PCR) and qPCR (RT-qPCR)

Total RNA was isolated from different tissues of mice. To analyze the expression of *Meiok21* mRNAs, cDNA was synthesized using the PrimeScript RT reagent Kit with gDNA Eraser (Takara). qPCR was performed using UltraSYBR Mixture (CWBiotech) and specific forward and reverse primers; the input of cDNA for each sample was 200 ng. *Meiok21* primers: (Forward, on exon 3) 5′-GGG AGG AAC CAC TAC ACG A-3′ and (Reverse, on exon 6) 5′- GCT TTG AGG G CT CCC AGC TC-3′. β-Actin was amplified as a housekeeping gene with the primers (Forward) 5′-CAT CCG TAA AGA CCT CTA TGC CAA C-3′ and (Reverse) 5′-ATG GAG CCA CCG ATC CAC A-3′. All PCR reactions were performed with an initial denaturation at 95°C for 10 min followed by 40 cycles of denaturation at 95°C for 30 s, annealing at 60°C for 30 s, extension at 72°C for 30 s and a final extension at 72°C for 5 min. The *Meiok21* expression level was quantified by the ΔCT method and the PCR products were analyzed on a 2% agarose gel.

To confirm *Meiok21* knockout efficiency, RT-PCR was performed using the same primers used for RT-qPCR. To detect possible transcripts in *Meiok21* knockout, another RT-PCR was performed using the following primers outside of the knockout region. Forward (on 5′ UTR): 5′-CAG AGC AAC TTC CAT TAC TGC-3′ and Reverse (on 3′UTR): 5′-ACA AAC TCA ACG ACA ACT GC-3′. The RT-PCR reaction was performed with an initial denaturation at 95°C for 3 min followed by 35 cycles of denaturation at 95°C for 30 s, annealing at 60°C for 30 s, extension at 72°C for 30 s and a final extension at 72°C for 5 min. The PCR products were analyzed on a 2% agarose gel and sequenced.

### Statistical analysis

Statistical analyses were conducted using GraphPad PRISM version 5.01 (GraphPad Software, Inc. RRID:SCR_002798). The error bars show means ± SEM or means ± SD as indicated in figure legends. Sample sizes were described in figure legends. The statistical significance of the differences between two sets of data was measured by Student's *t*-test with an unpaired, two-tailed distribution. The levels of significance are indicated as follows: *P* < 0.05 (*), *P* < 0.01 (**) and *P* < 0.001 (***).

## RESULTS

### MEIOK21 specifically localizes on meiotic chromosomes

**Figure 1.**
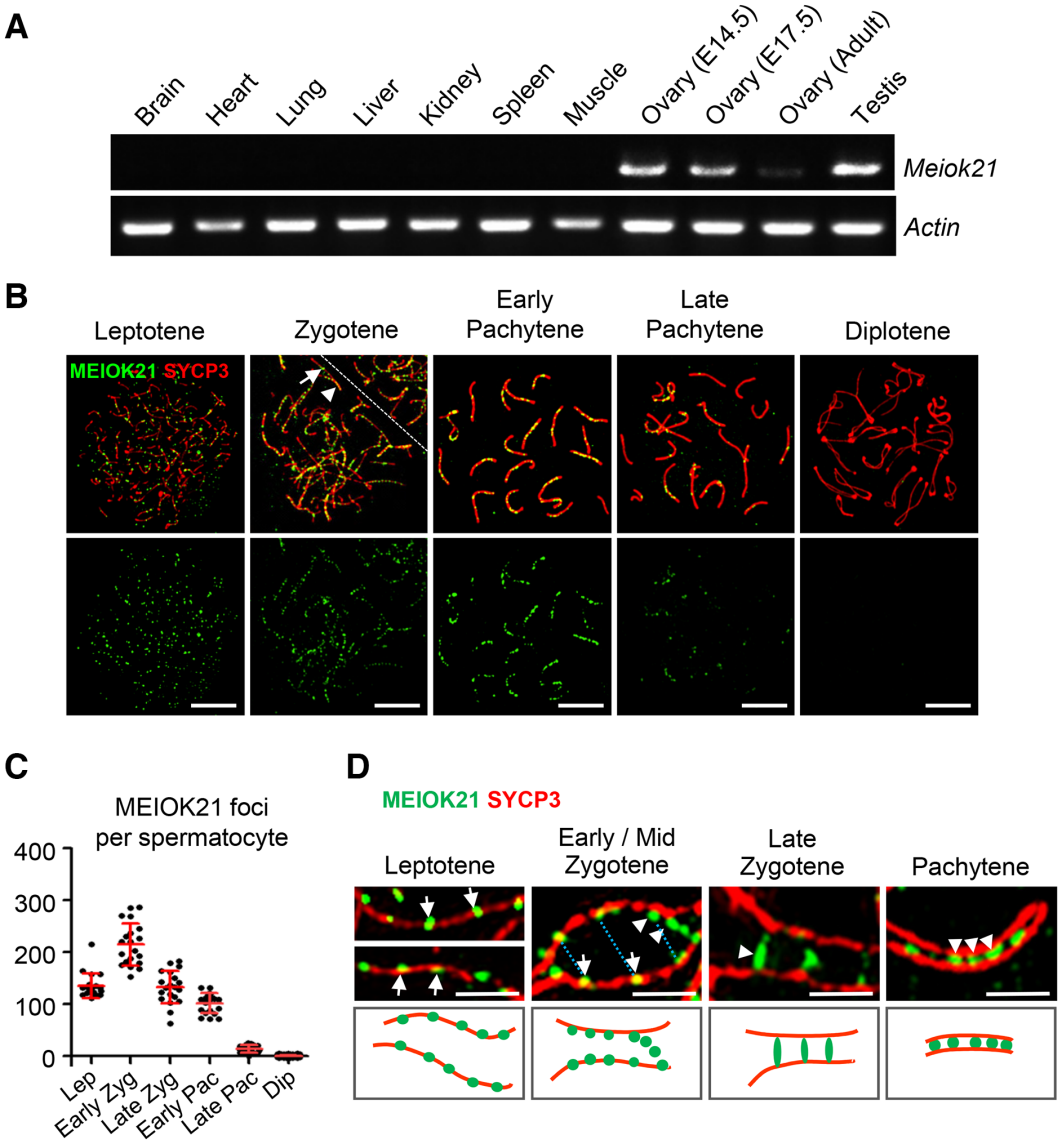
Dynamic localization of MEIOK21 on meiotic chromosomes. (**A**) *Meiok21* is highly expressed in testes and fetal ovaries, revealed by RT-qPCR. (**B**) The dynamic localization of MEIOK21 to meiotic chromosomes in mouse spermatocytes. The meiotic stages of spermatocytes were determined by the SYCP3 staining (red) of the chromosome axis. MEIOK21 (green) are visualized on meiotic chromosomes from leptotene to late pachytene and are undetectable in diplotene. The arrow in zygotene indicates MEIOK21 foci on an unsynapsed axis, 77.60 ± 8.24% in early/mid zygotene and 26.08 ± 8.98% in late zygotene. The arrowhead indicates MEIOK21 foci on a synapsed axis, 22.40 ± 8.24% in early/mid zygotene and 73.92 ± 8.98% in late zygotene. Mean ± SD, *n* = 20 for each group. The dashed line separates the target cell from the adjacent unrelated cell. Scale bar, 10 μm. (**C**) Quantification of the numbers of MEIOK21 foci in spermatocytes from leptotene to diplotene. Spermatocyte sample sizes, *n* = 20 for each stage. Lep, leptotene; early Zyg, early zygotene; late Zyg, late zygotene; early Pac, early pachytene; late Pac, late pachytene; Dip, diplotene. Error bar, mean ± SD. (**D**) The dynamic localization of MEIOK21 on chromosomes investigated by structured illumination microscope (SIM). (Top panel) MEIOK21 foci first localize on the chromosome axis (‘leptotene’, arrows). Then, on chromosome regions where two homolog axes are aligned in parallel, MEIOK21 foci were frequently seen as ‘pairs’ at the opposing sites on the two homologs (‘early/mid zygotene’, arrows, MEIOK21 foci; blue dotted lines, focus pairs) and some foci are released as ‘hanging foci’ (‘early/mid zygotene’, arrowheads). When homologous chromosomes aligned closely, MEIOK21 appears as ‘bridges’ or ‘fusing foci’ (‘late zygotene’, arrowhead) and finally as fused foci (‘pachytene’, arrowheads) between synapsed axes. (Bottom panel) cartoons illustrate the dynamic localization of MEIOK21 with meiotic progression. Scale bar, 1 μm.

To understand the mechanisms of meiotic recombination, RNA sequencing of mouse germ cells was conducted to identify new factors involved in meiotic DSB repair and recombination (33). An uncharacterized gene *4930432K21RIK* was selected based on its preferential expression in germ cells during meiosis, which was also previously identified as being highly expressed in mouse testes (38). This gene is 44.57 kb, located on chromosome 8. It contains nine exons with start codon ATG in the second exon and stop codon TAA in the last exon, and the ORF is 1,800 bp, encoding 600 amino acids. By searching its sequence in public databases, including NCBI, Uniprot and Protein Data Bank, we found that 4930432K21RIK protein only exists in vertebrates and is rather conserved (Supplementary Figure S1A). It belongs to an unknown protein superfamily (cl25823), which contains a conserved structural domain with unknown function (DUF4671; https://www.ncbi.nlm.nih.gov/Structure/cdd/cddsrv.cgi?uid=318011). Its human ortholog is C19ORF57, which is also preferentially expressed in testes (39). To further study the expression patterns of *4930432K21RIK*, reverse transcription qPCR (RT-qPCR) was performed. We found that mRNA was highly expressed in mouse testes and fetal ovaries (Figure 1A and Supplementary Figure S1BC). According to its biological function described below, this gene was named *Meiok21* (**meio**sis-specific *4930432****K21****RIK*).

Given that *Meiok21* is highly expressed in testes and fetal ovaries, where meiosis occurs, the expression and localization of MEIOK21 in spermatocytes was further examined in detail by chromosome spread combined with immunostaining. Meiotic stages were classified according to standard cytological criteria combined with the morphology of SYCP3, a chromosome axis marker (40). At the leptotene stage, developing chromosome axes are labelled by short stretches of SYCP3. At the zygotene stage, these discontinuous SYCP3 stretches become linear axes and homologs begin to synapse where the SYCP3 labelled axes are much thicker than unsynapsed regions (Figure 1B). When all autosomes completely synapsed, the nucleus enters the pachytene stage. After pachytene, synaptonemal complexes (SCs) gradually disassemble and the nucleus enters the diplotene stage. Finally, SCs disappear from chromosomes except at a few sites where chiasmata hold homologs together (41). The last stage of meiotic prophase I is diakinesis, when SYCP3 axes break down except at centromeres (41).

MEIOK21 was first observed on chromosome axes with ∼135 foci at leptotene in spread spermatocytes immunostained with anti-MEIOK21 antibodies (Figure 1B). The number of MEIOK21 foci increased to a peak of ∼200 at early zygotene. After that, the number of MEIOK21 foci gradually decreased. At late pachytene, only a small number of MEIOK21 foci were observed and they were undetectable at diplotene when SC began to disassemble (Figures 1B and C). However, MEIOK21 foci were found on both synapsed and unsynapsed chromosome regions (Figure 1B, arrow vs arrowhead). At early-mid zygotene, ∼77% of MEIOK21 foci were on unsynapsed regions and ∼23% were on synapsed regions. With meiosis progression, MEIOK21 focus number decreased to ∼26% on unsynapsed regions and increased to ∼74% on synapsed regions at late zygotene. Therefore, MEIOK21 dynamically localizes on meiotic chromosomes during prophase of meiosis I.

The dynamic localization pattern of MEIOK21 foci on meiotic chromosomes is quite similar to many well-established recombination factors such as RPA and RAD51/DMC1 recombinase (42–44). These observations prompted us to examine the localization of MEIOK21 foci more extensively. Using structured illumination microscopy (SIM), we found that MEIOK21 was located on the chromosome axis as foci at leptotene (Figure 1D left, arrows). At zygotene, on chromosome regions where homologous chromosomes were still widely separated, MEIOK21 foci localized on the axis as at leptotene (Figure 1D middle left, arrows), while on chromosome regions where two homolog axes were aligned in parallel (800–1000 nm), MEIOK21 foci were frequently seen as ‘pairs’ at the opposing sites on the two homologs (Figure 1D top, middle left, blue dotted lines), and many MEIOK21 foci were seen as ‘hanging foci’, attached to the sides of axes (Figure 1D middle left, arrowheads).

At the more advanced chromosome regions (homolog axes spaced at 300–700 nm), MEIOK21 foci were seen as ‘bridge’ like structures or ‘fusing foci’, which is quite common in late zygotene spermatocytes (Figure 1D middle right, arrowhead). At pachytene, when all autosomes were synapsed, a majority (∼70%, *n* = 1192 foci) of MEIOK21 foci were located between the synapsed axes, most probably on SC complexes (Figure 1D right, arrowheads), although there were still some MEIOK21 foci that remained on the axis or as hanging foci. This progression (Figure 1D, lower panels), from on-axis foci to ‘hanging foci’, then to a ‘bridge’ like structure and finally to single foci between axes, has been proved to reflect typical dynamics of recombination factors, such as RPA in humans and Msh4 in *Sordaria*, during the meiotic recombination process (31,45,46). Taken together, the distribution patterns of MEIOK21 coincide with typical features of recombination factors, suggesting its potential roles in meiotic recombination.

### MEIOK21 localizes to meiotic recombination sites in a DSB-dependent manner

**Figure 2.**
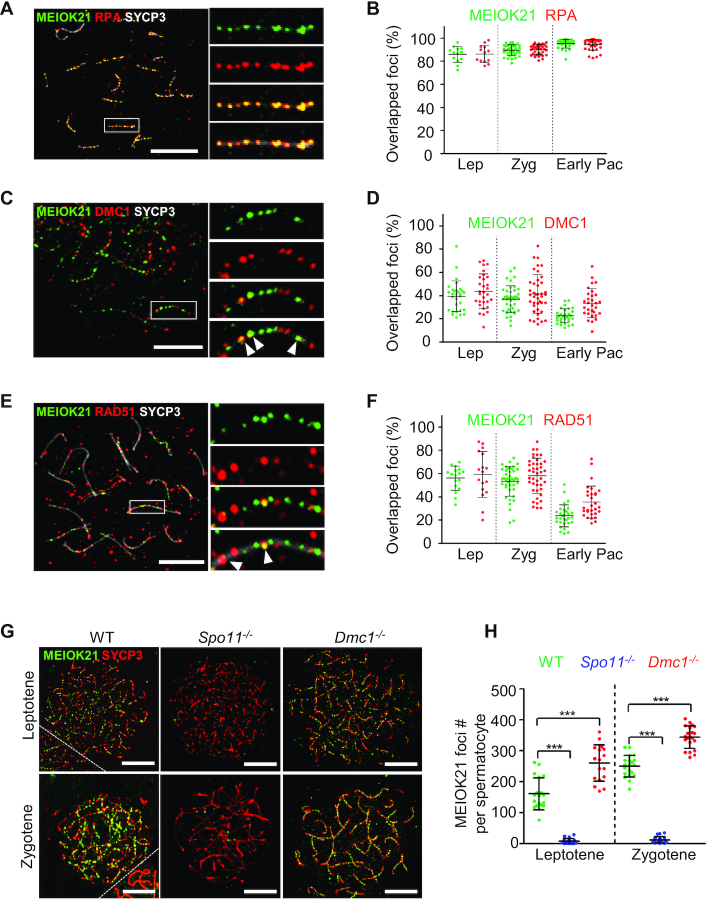
MEIOK21 localizes to meiotic recombination sites in a DSB dependent manner. (**A**) MEIOK21 colocalizes with RPA. (**B**) Quantification shows that ∼85-95% of the two factors overlap with each other from leptotene to early pachytene. Error bar, mean ± SD. Sample sizes (from left to right), *n* = 13, 13, 37, 37, 30 and 30, respectively. (**C**) MEIOK21 partially colocalizes with DMC1. (**D**) From leptotene to zygotene, ∼40% of the two factors overlap with each other; in early pachytene, ∼20% of MEIOK21 foci overlap with DMC1 foci, and ∼30% of DMC1 foci overlap with MEIOK21 foci. Error bar, mean ± SD. Sample sizes (from left to right), *n* = 30, 30, 41, 41, 30 and 30, respectively. Arrowheads indicate overlapped foci. (**E**) MEIOK21 partially colocalizes with RAD51. (**F**) From leptotene to zygotene, ∼60% of the two factors overlap with each other; in early pachytene, ∼20% of MEIOK21 foci overlap with RAD51 foci, and ∼30% of RAD51 foci overlap with MEIOK21 foci. Error bar, mean ± SD. Sample sizes (from left to right), *n* = 17, 17, 42, 42, 30 and 30, respectively. Arrowheads indicate overlapped foci. (**G**) MEIOK21 foci are abolished in *Spo11^−^^/^^−^* spermatocytes, but more MEIOK21 foci are observed in *Dmc1^−^^/^^−^* spermatocytes compared to WT. Note that *Spo11^−^^/^^−^* and *Dmc1^−^^/^^−^* spermatocytes can only reach the ‘zygotene’ stage. The dashed line separates the target cell from the adjacent unrelated cell. Scale bar, 10 μm (**A, C, E, G**). (**H**) The quantification of MEIOK21 foci in spermatocytes of WT, *Spo11^−^^/^^−^* and *Dmc1^−^^/^^−^* mice. Spermatocyte sample sizes, from left to right, n = 19, 22, 17, 20, 22 and 21. Error bar, mean ± SD.

The uniquely dynamic localization of MEIOK21 during meiosis strongly indicates it may function in meiotic recombination. To further investigate this, MEIOK21 was co-immunostained separately with known recombination factors RAD51, DMC1 and RPA protein component (RPA2). We found that MEIOK21 foci colocalized with RPA foci at a high frequency, ∼85-95% of the two factors overlapped with each other from leptotene to early pachytene (Figure 2AB). Partial colocalizations were found between MEIOK21 and DMC1 or RAD51. From leptotene to zygotene, ∼40% of MEIOK21 and DMC1 foci overlapped with each other. At early pachytene, ∼20% of MEIOK21 foci overlapped with DMC1 foci, and ∼30% of DMC1 foci overlapped with MEIOK21 foci (Figure 2CD). Similar situations were found between MEIOK21 and RAD51. From leptotene to zygotene, ∼60% of MEIOK21 and RAD51 foci overlapped with each other; at early pachytene, ∼20% of MEIOK21 foci overlapped with RAD51 foci, and ∼30% of RAD51 foci overlapped with MEIOK21 foci (Figure 2EF).

The proper localization of recombination factors, including RPA, RAD51 and DMC1, depends on programmed DSBs catalyzed by the SPO11 complex (47). Thus, we checked whether the localization of MEIOK21 on chromosomes was DSB dependent or not. In WT, there were abundant MEIOK21 foci located on chromosomes in both leptotene and zygotene nuclei (Figure 2G left and 2H). However, MEIOK21 foci were rarely observed in *Spo11^−^^/^^−^* spermatocytes (Figure 2G middle and H). This supports that the chromosome localization of MEIOK21 depends on meiotic DSBs and MEIOK21 may play important roles in DSB repair. In *Dmc1^−^^/^^−^* spermatocytes, DSBs are unrepaired and accumulated with extensive resection (48). The localization of MEIOK21 was further examined in *Dmc1^−^^/^^−^* spermatocytes. Consistent with this, the number of MEIOK21 foci was also accumulated to a significantly higher level (Figure 2G right versus left; H). Therefore, the localization of MEIOK21 on chromosomes depends on meiotic DSBs and occurs prior to DMC1.

### MEIOK21 interacts with HSF2BP

**Figure 3.**
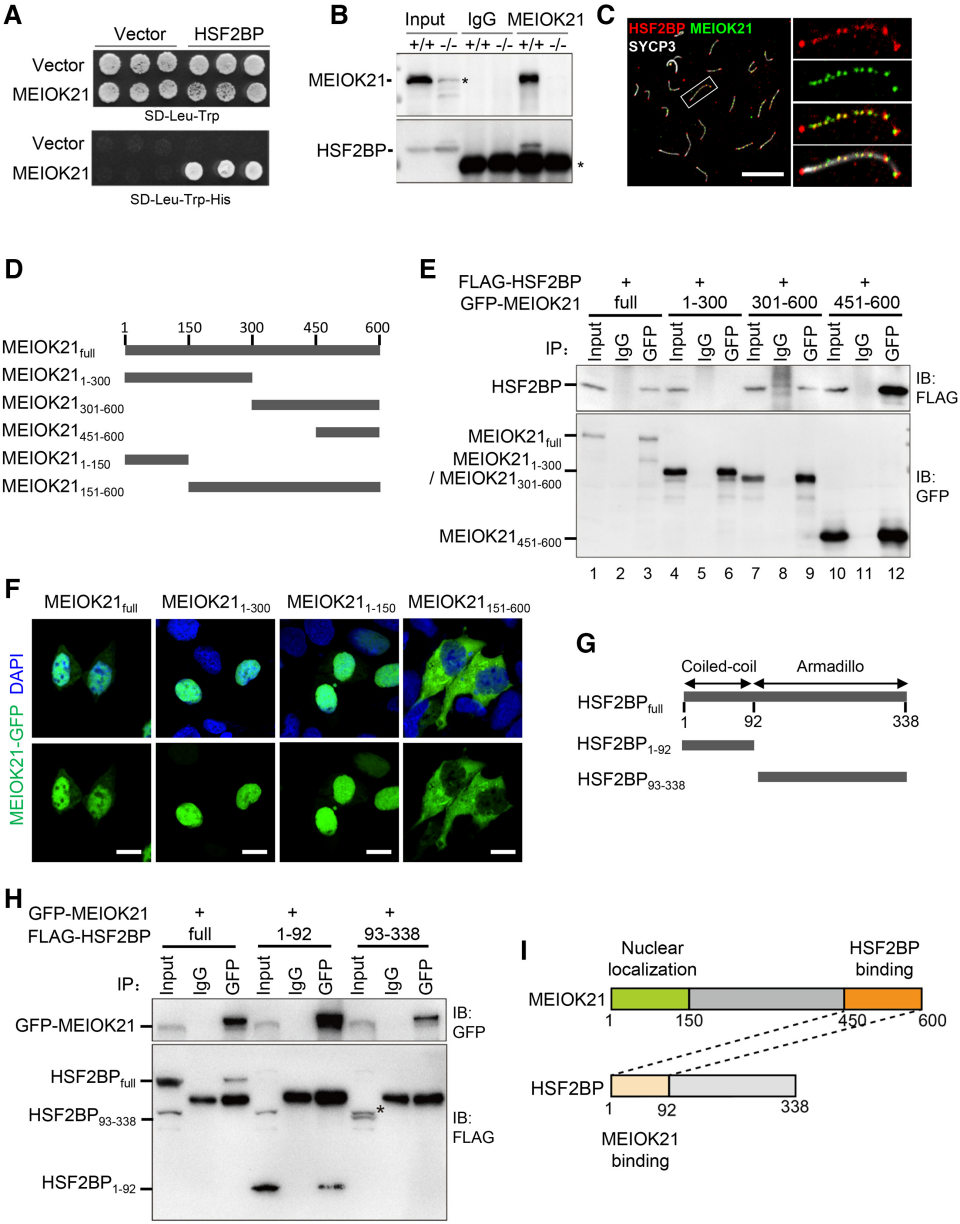
MEIOK21 interacts with recombination factor HSF2BP. (**A**) MEIOK21 interacts with HSF2BP in Y2H screening, with MEIOK21 as prey and HSF2BP as bait. About 10^5^ yeast cells were seeded in each dot. The experiment was repeated three times. (**B**) HSF2BP is pulled-down in *Meiok21^+/+^* testis lysate (+/+) (200 mg) but not *Meiok21^−^^/^^−^* testis lysate (−/−) (200 mg) by antibody against MEIOK21.The input amount is ∼5% of the lysate. IgG acts as a negative control. The experiment was repeated three times. Nonspecific bands are labeled with *. (**C**) MEIOK21 colocalizes with HSF2BP on meiotic chromosomes (see Supplementary Figure 2E for quantifications). Scale bar, 10 μm. (**D**) Diagram of MEIOK21 truncations. (**E**) The C terminus of MEIOK21 (a.a. 451–600) is required for interaction with HSF2BP. FLAG-tagged HSF2BP and GFP-tagged MEIOK21 truncations were co-transfected to HEK-293T cells. After 48 hours, cells were lysed, and co-IP experiments were performed using GFP antibody. The input amount is ∼5% of the lysate. The experiment was repeated twice. (**F**) The N terminus of MEIOK21 (a.a. 1–150) is required for its nuclear localization. GFP-tagged MEIOK21 truncations were transfected to HEK-293T cells. After 24 hours, GFP signal was examined under a fluorescence microscope. Scale bar, 5 μm. (**G**) The diagram of HSF2BP truncations. (**H**) The coiled-coil domain of HSF2BP (a.a. 1–92) is required for MEIOK21 binding. GFP-tagged MEIOK21 and FLAG-tagged HSF2BP truncations were co-transfected to HEK-293T cells. After 48 hours, cells were lysed, and co-IP experiments were performed with antibodies against GFP. The input amount is ∼5% of the lysate. The experiment was repeated twice. The non-specific band (*). (**I**) Diagrams to illustrate interaction domains of MEIOK21 and HSF2BP.

To clarify the possible function of MEIOK21 during meiotic recombination, we searched for its potential interactors during this process. We focused on RPA and factors that also colocalize with RPA. In mammals, the RPA complex is composed of RPA1, RPA2 and RPA3. Recently, MEIOB, SPATA22 and HSF2BP have been found to colocalize with RPA, and they are all required for meiotic recombination (26–28,30,49,50). Thus, these proteins were screened for their possible interactions with MEIOK21 using a yeast two-hybrid (Y2H) assay. Among them, only HSF2BP was detected to interact with MEIOK21 (Figure 3A; Supplementary Figure S2A-C). To further confirm the interaction between MEIOK21 and HSF2BP, endogenous co-immunoprecipitation (co-IP) assays were performed using WT testis extracts. Consistent with the Y2H result, endogenous HSF2BP could be pulled down by MEIOK21 in co-IP via anti-MEIOK21 antibody. As a negative control, HSF2BP was not detected in co-IP using *Meiok21^−^^/^^−^* testis extracts in which MEIOK21 protein is absent (Figure 3B). Consistent with the interaction results, ∼75–90% of MEIOK21 foci colocalized with HSF2BP foci, and ∼70–90% of HSF2BP foci colocalized with MEIOK21 foci on meiotic chromosomes from leptotene to pachytene (Figure 3C and Supplementary Figure S2E). Above results support that MEIOK21 binds to HSF2BP *in vivo*, and functions in meiotic recombination.

Next, we asked which region(s) of MEIOK21 interact(s) with HSF2BP. Since the full- length protein is composed of an unknown structural domain (DUF4671), without any other known functional domains/motifs, several GFP tagged truncations were constructed (Figure 3D). We found the GFP-tagged MEIOK21_301-600_ fragments pulled down Flag-tagged HSF2BP as efficiently as full length MEIOK21, while MEIOK21_1–300_ fragments could not pull down HSF2BP (Figure 3E, lane 9 versus lane 6). This indicates that the MEIOK21 C-terminal but not the N-terminal fragment is responsible for its interaction with HSF2BP. Further experiments showed that the last 150 amino acids (MEIOK21_451–600_) were sufficient to interact with HSF2BP (Figure 3E, lane 12).

Given that MEIOK21 localized in the nucleus (specifically on meiotic chromosomes), but bioinformatic analysis did not identify a nuclear localization sequence, we wondered which region of MEIOK21 mediated its nucleus localization. For this purpose, we first confirmed GFP-tagged full length MEIOK21 protein localized to the nucleus when it was transiently expressed in HEK-293T cells (Figure 3F). Then, GFP-tagged MEIOK21 fragments (above) were transfected into HEK-293T cells. The N-terminal fragment containing the first 150 amino acids localized to the nucleus efficiently (Figure 3F), which suggests that this fragment contains the nuclear localization signal. However, the fragment lacking the first 150 amino acids, MEIOK21_151–600_, localized to the cytoplasm exclusively (Figure 3F). Therefore, MEIOK21 has at least two functional domains, the N-terminal 150 amino acids for nuclear localization and the C-terminal 150 amino acids for interaction with HSF2BP.

HSF2BP has two domains, the N-terminal coiled-coil domain interacting with HSF2 and the C-terminal armadillo domain interacting with BRCA2 and BNC1 (28,51,52). Our Y2H and co-IP experiments show that the N-terminal domain of HSF2BP but not the C-terminal domain can sufficiently mediate its interaction with MEIOK21 (Figures 3G–I and Supplementary Figure S2D). However, it is still unknown whether MEIOK21 and HSF2 share the same binding motif at the N-terminus of HSF2BP, and how these two factors interact with and regulate HSF2BP. These questions remain to be further investigated.

### MEIOK21 is required for male fertility

**Figure 4.**
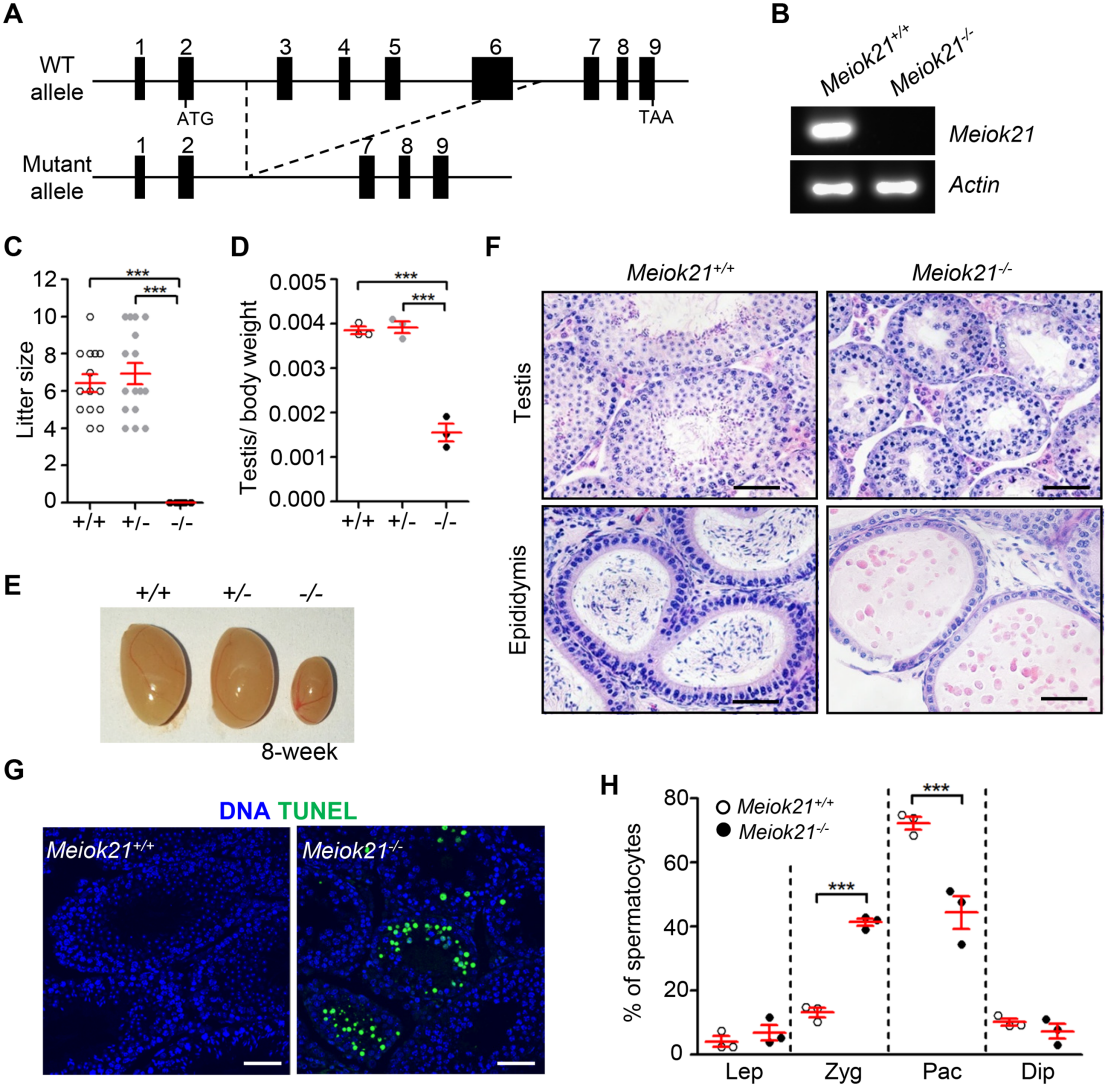
MEIOK21 is required for male fertility. (**A**) The schematic of *Meiok21* knockout allele. Exons 3–6 were deleted in the mutant. (**B**) *Meiok21* knockout was confirmed by RT-PCR (Materials and Methods). RT-PCR was performed using cDNA obtained from testes as templates. PCR products with proper size were detected from WT but not mutant samples. (**C**) *Meiok21^−^^/^^−^* male mice were sterile. *n* = 5 for each genotype. Error bar, mean ± SEM. (**D**, **E**) Both the testis size and testis/body weight ratio of 8-week-old *Meiok21^−^^/^^−^* mice are significantly reduced compared to *Meiok21^+/+^*and *Meiok21^+/^^−^* mice. Error bar, mean ± SEM. *n* = 3 for each genotype. (**F**) No spermatids or mature spermatozoa were observed in testis or epididymis by HE staining in *Meiok21^−^^/^^−^* mice. Scale bar, 50 μm. (**G**) A large number of apoptotic spermatocytes (green) were observed in *Meiok21^−^^/^^−^* testis by TUNEL staining. Scale bar, 50 μm. (**H**) Quantitative analysis of spermatocytes at different meiotic stages from *Meiok21^+/+^* and *Meiok21^−^^/^^−^* mice testes at postnatal day 20. There are more zygotene but less pachytene spermatocytes in *Meiok21^−^^/^^−^* testis compared to WT. *n* = 3 for each genotype. Error bar, mean ± SEM.

MEIOK21 specifically localizes on chromosomes in a DSB dependent manner. It colocalizes with RPA and interacts with HSF2BP, which is involved in meiotic recombination. All of these traits strongly suggest that MEIOK21 likely functions directly in meiosis. To test this, *Meiok21* deficient (*Meiok21^−^^/^^−^*) mice were generated by deleting exons 3–6 of *Meiok21* using the CRISPR/Cas9 system (Figure 4A and Supplementary Figure S3A). The knockout efficiency was confirmed by RT-PCR and immunostaining against endogenous MEIOK21 (Figures 4B and Supplementary Figure S3B–F). *Meiok21^−^^/^^−^* mice were viable and appeared to develop normally. Although *Meiok21^−^^/^^−^* female mice had no obvious fertility defects, *Meiok21^−^^/^^−^* male mice were infertile (Figure 4C, Supplementary Figure S4A). Studies showed that the sizes of testes from 8-week-old *Meiok21^−^^/^^−^* male mice were much smaller than those of WT and *Meiok21^+/^^−^* mice at the same age (Figure 4D, E). However, 12-week-old *Meiok21^−^^/^^−^* ovaries were still comparable to WT (Supplementary Figure S4B, C). Further analysis showed no post-meiotic round spermatids or elongating spermatids in the seminiferous tubules, but there were numerous vacuoles in *Meiok21^−^^/^^−^* testes, which were rarely seen in WT testes (Figure 4F, upper panel). Moreover, no spermatozoa were found in the cauda epididymis of *Meiok21^−^^/^^−^* mice, whereas mature spermatozoa were present in the cauda epididymis of WT (Figure 4F, lower panel). TdT-mediated dUTP nick end labeling (TUNEL) staining of *Meiok21^−^^/^^−^* testis sections revealed that many spermatocytes underwent apoptosis (Figure 4G). These results suggest that in the absence of MEIOK21, a large number of spermatocytes are eliminated before the completion of spermatogenesis, which results in smaller testes as often seen in synapsis- and recombination-defective mutants, such as *Dmc1^−^^/^^−^* and *Msh5^−^^/^^−^* (44,53,54). Therefore, MEIOK21 is essential for spermatogenesis and male fertility.

### MEIOK21 is important for normal meiosis progression

Given that *Meiok21^−^^/^^−^* spermatocytes are eliminated via apoptosis, we speculate that they may undergo severe defects in meiosis. Spermatogenesis in mice is a continuous process and occurs by waves in seminiferous tubules. Thus, spermatocytes at different stages can be seen in a single seminiferous tubule. The first wave of spermatogenesis is relatively synchronized (55). The stage(s) in which the defects occur can be easily inferred from meiosis progression. Spermatocytes from leptotene to diplotene can be distinguished based on chromosome immunostaining of SYCP3 and SYCP1, the components of meiotic chromosome axis and SC. Zygotene spermatocytes were dominant in postnatal day 15 (PD15) testis of both WT and *Meiok21^−^^/^^−^* mice, and there were slightly more zygotene (∼70% vs ∼65%) and significantly less pachytene (∼7% versus ∼16%) spermatocytes in *Meiok21^−^^/^^−^* compared to WT. With meiosis progression, more zygotene cells entered pachytene, thus the frequency of zygotene cells decreased, and concomitantly, the frequency of pachytene cells increased. As expected, at PD17, zygotene spermatocyte frequency decreased to ∼25% and pachytene spermatocyte frequency increased to ∼61% in WT. However, ∼64% of spermatocytes stayed in zygotene and only ∼22% of spermatocytes entered pachytene in *Meiok21^−^^/^^−^* mice. At PD20, zygotene spermatocytes further decreased to ∼15% and pachytene spermatocytes increased to ∼72% in WT. But in *Meiok21^−^^/^^−^*, zygotene spermatocytes were still at a high level (∼40%) and pachytene spermatocytes only increased to ∼45%, (Figure 4H). Therefore, there were always significantly more zygotene spermatocytes and fewer pachytene spermatocytes in *Meiok21^−^^/^^−^* mice, compared to WT at the same ages (Figure 4H). These observations suggest that in the absence of MEIOK21, meiosis might be arrested or delayed at the zygotene/pachytene transition, and only a subset of spermatocytes that have fewer defects could enter pachytene.

Since no spermatids were found in *Meiok21^−^^/^^−^* testis, we focused on seminiferous epithelium to examine the pathological status of spermatogenesis in *Meiok21^−^^/^^−^* testes. Mouse spermatogenesis is a highly ordered biological process, and the seminiferous epithelium can be divided into twelve stages (stages I–XII) according to the development of spermatocytes and spermatids, which can be visualized by the periodic acid-Schiff's reaction (PAS) (55). In WT testis, seminiferous tubules from stage I–XII can be observed, and spermatocytes from leptotene to metaphase I, together with round spermatids and elongating spermatids, are organized in corresponding positions (Supplementary Figure S5A; upper panel). In *Meiok21^−^^/^^−^* testis, spermatids were never found, and stages of seminiferous epithelium could only be identified based on the types of spermatocytes (Supplementary Figure S5A; lower panel) (55). Moreover, in *Meiok21^−^^/^^−^* tubules, apoptotic spermatocytes were observed as early as stage IV and throughout stages after that. At stage IV, pachytene spermatocytes are dominant in WT tubules, however, many apoptotic pachytene spermatocytes with aberrantly condensed nuclei appeared in *Meiok21^−^^/^^−^* tubules (55) (Supplementary Figure S5A; lower panel, ‘aP’). These results suggest that defective *Meiok21^−^^/^^−^* pachytene spermatocytes may undergo apoptosis. In addition, apoptotic diplotene (aDi) and apoptotic metaphase I (aMI) spermatocytes were also found in *Meiok21^−^^/^^−^* tubules (Supplementary Figure S5A; lower panel, ‘aDi’ and ‘aMI’). Very similar results were observed in both younger and older mice (Supplementary Figure S5BC). We speculated that a small subset of *Meiok21^−^^/^^−^* spermatocytes with fewer defects could bypass the pachytene checkpoint, but finally undergo apoptosis. Therefore, defective *Meiok21^−^^/^^−^* spermatocytes probably are cleared via apoptosis during prophase of meiosis I, which results in male infertility.

### *Meiok21^−^^/^^−^* spermatocytes show defects in synapsis and crossover recombination

**Figure 5.**
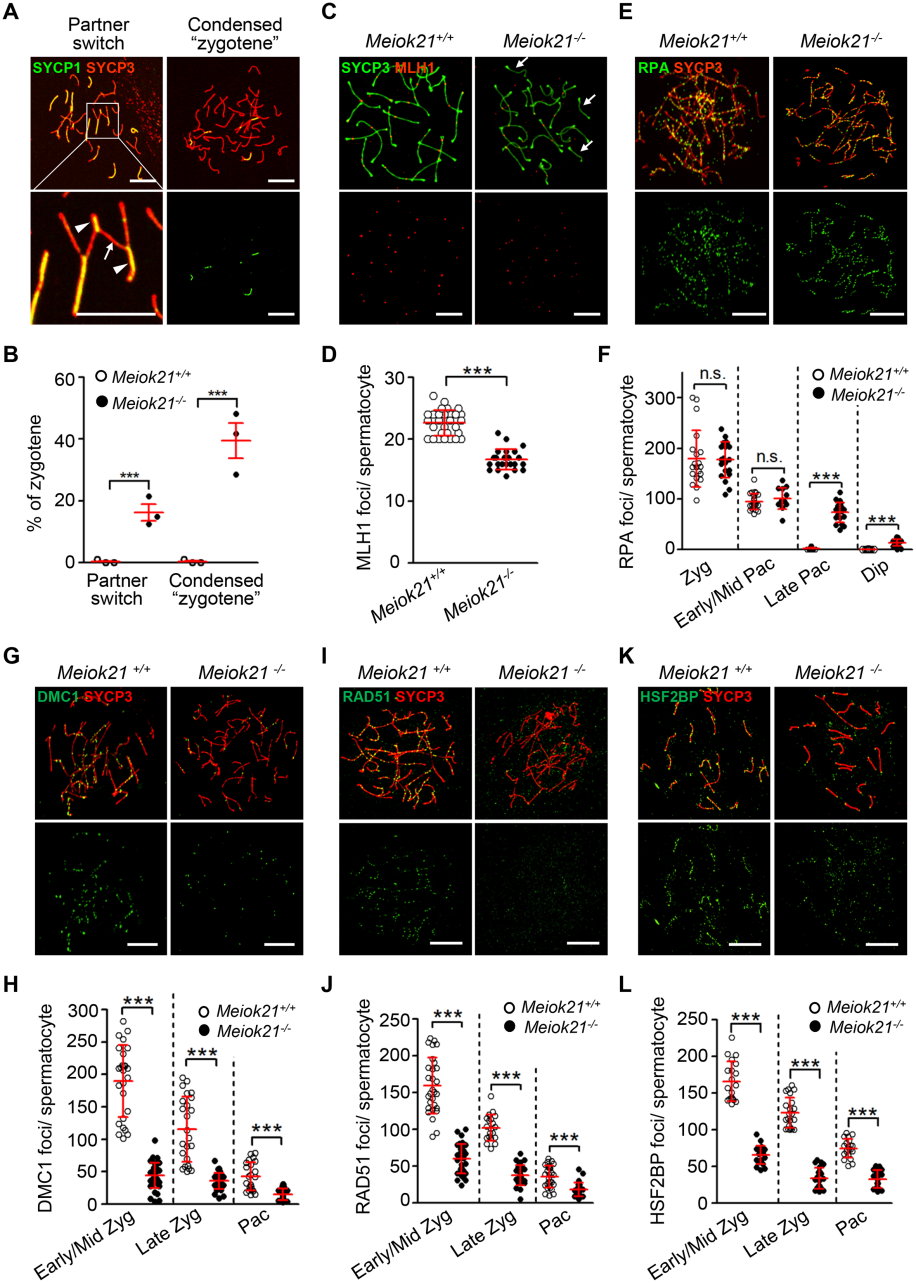
MEIOK21 is required for efficient meiotic recombination. (**A**) Two types of aberrant synapsis are frequently observed in *Meiok21^−^^/^^−^* spermatocytes, as seen in other recombination defective mutants. Partner switch (left panel), i.e. one chromosome (arrow) synapses with more than one partner (arrowheads). (**B**) Quantification of the two types of aberrant ‘zygotene’ nuclei described in (A). For each genotype, 3 mice were examined. The numbers of cells examined in each mouse are 84, 60 and 60 for WT, and 93, 48 and 64 for *Meiok21^−^^/^^−^*. (**C**, **D**) The number of crossovers, marked by MLH1 foci (red), is significantly reduced in *Meiok21^−^^/^^−^* pachytene spermatocytes compared to WT. *n* = 20 and 23 for WT and *Meiok21^−^^/^^−^*, respectively. Chromosomes without even one MLH1 focus (arrows). (**E**, **F**) In *Meiok21^−^^/^^−^* spermatocytes, the level of RPA foci is similar to that of WT at zygotene, but there is aberrant retention of RPA on chromosomes at a late time. From left to right, n = 20, 20, 24, 13, 23, 19, 14 and 20, respectively. (**G–L**) The numbers of DMC1, RAD51 and HSF2BP foci are greatly decreased in *Meiok21^−^^/^^−^* spermatocytes compared to WT. Early/Mid Zyg, early or middle zygotene; Late Zyg, late zygotene; Pac, pachytene, Late Pac: late pachytene, Dip: diplotene. For each panel from left to right, *n* = 26, 36, 24, 30, 22 and 27 (H), 29, 36, 20, 27, 29 and 31 (J), 20, 21, 22, 21, 21 and 21 (L). Scale bar, 10 μm (A, C, E, G, I, K). Error bar, mean ± SD (D, F, H, J, L) or mean ± SEM (B).

The phenotype of *Meiok21^−^^/^^−^* mice is similar to many synapsis or recombination defective mutants (56,57). Thus, homologous synapsis and meiotic recombination were examined in *Meiok21^−^^/^^−^* spermatocytes. By double staining of SYCP3 and SYCP1, two types of synapsis defects were found in *Meiok21^−^^/^^−^* spermatocytes that were never seen in WT mice. (i) Synapsis partner switch, i.e. different regions of one chromosome synapses with more than one partner (Figure 5A, left panel). In this case, at least one partner is a non-homolog, meaning there is also non-homologous synapsis, which has been reported in several recombination mutants in mice (27,30,44). (ii) Condensed ‘zygotene’. There is a fraction of ‘zygotene’ nuclei with highly condensed chromosomes as pachytene nuclei, in which there is no or only few short SYCP1 stretches. Thus, in these nuclei homologs are unsynapsed and remained as ∼40 ‘univalents’ but not 20 bivalents as seen in WT pachytene (Figure 5A, right panel). This type of synapsis defect is often seen in recombination defective mutants (7,40,44,53). In total, these two types of abnormal nuclei account for ∼50% of the zygotene spermatocytes in *Meiok21^−^^/^^−^* mice (Figure 5B). Such abnormalities are not found in *Meiok21^−^^/^^−^* oocytes (Supplementary Figure S4D). We infer that these nuclei are unable to enter the pachytene stage. This supports the idea that the high level of zygotene nuclei in the mutants is not due to a delay in entering pachytene, but actually due to a fraction of nuclei that permanently arrest in the zygotene stage because of severe synapsis failures. However, we cannot exclude the possibility that these aberrant nuclei are actually at a pachytene-like stage but with pairing/synapsis defects.

As described above, a considerable proportion of *Meiok21^−^^/^^−^* spermatocytes can still enter pachytene. In order to characterize whether these pachytene nuclei are normal, meiotic recombination was examined. The final and major purpose of meiotic recombination is the formation of homologous DNA crossovers (COs). COs cause the exchange of homologous DNA and establish the physical connections between homologs required for proper chromosome segregation (12). Around 90% of mouse COs can be marked by MLH1 foci at pachytene (58–60). So, the number and distribution of MLH1 foci were checked by immunostaining in pachytene spermatocytes. Consistent with previous studies, in WT pachytene spermatocytes, ∼22 MLH1 foci were observed per cell and almost every autosome has at least one MLH1 focus, thus autosomes without an MLH1 focus are rare (∼1%). Only ∼17 MLH1 foci were observed per *Meiok21^−^^/^^−^* pachytene spermatocyte, with ∼25% autosomes without even one MLH1 focus (Figures 5CD, Supplementary Figure S6A).

### MEIOK21 is required for normal DMC1 and RAD51 recruitment in spermatocytes

Meiotic crossover recombination originates from programmed DSBs. Upon DSB formation, γH2AX, the phosphorylated form of the histone variant H2AX, appears and its level is correlated with the level of DSBs (61,62). In WT spermatocytes, abundant γH2AX signals could be detected in the leptotene stage immediately after DSBs formed (Supplementary Figure S6B, upper two panels). In early pachytene nuclei, these signals decrease on autosomes, and bright signals appear on the XY body (63). From mid/late pachytene to diplotene, γH2AX signals are barely detectable on autosomes, however, strong signals are still maintained on the XY body. In *Meiok21^−^^/^^−^* spermatocytes, γH2AX signals were comparable to WT at leptotene and zygotene, suggesting that a WT level of DSBs are formed. Similar to WT, γH2AX signals also decreased on autosomes in early pachytene nuclei. However, in contrast to WT, a number of γH2AX foci were still maintained on autosomes at mid/late pachytene (45.66 ± 12.41 foci, *n* = 38) and even at diplotene (33.15 ± 14.73 foci, *n* = 47) (Supplementary Figure S6B, lower two panels). This implies that a considerable number of meiotic DSBs remained unrepaired in *Meiok21^−^^/^^−^* spermatocytes, though it seems chromosomes synapsed appropriately in these nuclei.

Meiotic DSBs are processed to generate long single strand 3′ overhangs, to which RPA binds (64). RPA foci appeared from leptotene and the number peaked at zygotene with a similar level in both WT and *Meiok21^−^^/^^−^* spermatocytes (179.60 ± 12.55 versus 177.50 ± 7.98) (Figure 5E, F). This further confirms that MEIOK21 is not required for DSB formation, and it is also consistent with the fact that localization of MEIOK21 depends on DSBs but not the other way around (above). In WT spermatocytes, the number of RPA foci decreased to a very low level at early/mid pachytene and RPA signals are barely detectable in late pachytene. In *Meiok21^−^^/^^−^* spermatocytes, the number of RPA foci also decreased at pachytene, but were still maintained at a relatively high level (73.52 ± 19.11 foci, *n* = 20), and even at diplotene a considerable number of RPA foci could still be observed in many nuclei (13.6 ± 6.51 foci, *n* = 20) (Figure 5EF). The aberrant retention of RPA in later prophase I is consistent with the persistence of γH2AX signals at these stages, indicating insufficient DSB repair in *Meiok21^−^^/^^−^* spermatocytes.

In meiosis, most DSBs are repaired with homologous templates. Both DMC1 and RAD51 form a nucleoprotein filament on ssDNA of DSBs to help search and invade its homologous partner for successful recombination and synapsis (13,19,65). At zygotene, abundant DMC1 foci can be observed on chromosomes in WT spermatocytes, and along with DSB repair, the number of DMC1 foci gradually decreases. However, in *Meiok21^−^^/^^−^* spermatocytes, only a very low number of DMC1 foci were observed at zygotene (44.14 ± 19.35 in *Meiok21^−^^/^^−^* versus 189.81 ± 54.23 in WT; Figure 5GH). Similarly, the number of RAD51 foci was also greatly reduced in *Meiok21^−^^/^^−^* spermatocytes compared to WT at zygotene (60.06 ± 19.62 versus 159.48 ± 37.33; Figure 5I, J). Western blot showed that the expression levels of RAD51 and DMC1 proteins were comparable in *Meiok21^−^^/^^−^* mutant and WT (Supplementary Figure S6C). These results suggest that MEIOK21 does not affect the DMC1/RAD51 protein expression but is required for DMC1 and RAD51 loading to meiotic DSB ends or their stability on chromosomes. This finding is consistent with two facts described above: (1) MEIOK21 does not affect DSB formation or its level, and (2) DMC1 is not required for MEIOK21 localization to recombination sites.

Greatly reduced DMC1 focus number also suggest greatly reduced levels of inter-homolog recombination interactions in *Meiok21^−^^/^^−^* mice (∼20–40% of WT level based on DMC1/RAD51 focus number), which will result in recombination and synapsis defects (13,44). However, as described above, in *Meiok21^−^^/^^−^* pachytene nuclei, the number of MLH1 foci is still at ∼77% of the WT level, which is less affected than inter-homolog recombination interactions (CO precursors) as indicated by DMC1/RAD51 foci (20-40% of WT). This result suggests that CO homeostasis may still exist in *Meiok21^−^^/^^−^* spermatocytes. CO homeostasis reflects the fact that when the number of CO precursors is significantly altered, the number of COs can be maintained constantly or only affected less proportionally (66–68).

Two other recombination factors, MSH4 and RNF212, which act in the later stages of meiotic recombination, were also examined. The data showed that, without MEIOK21, both MSH4 and RNF212 focus numbers were reduced to ∼70% of WT (Supplementary Figure S7A–D), which is comparable to the level of MLH1 foci (∼77% of WT), but much higher than the level of DMC1/RAD51 foci (20–40% of WT). This may explain why a considerable number of MLH1 foci can be formed in *Meiok21^−^^/^^−^* spermatocytes, and may also suggest that CO homeostasis (and thus CO/NCO differentiation) occurs at or before MSH4 foci occurrence as previously proposed (15,61,65,68).

### MEIOK21 is essential for sufficient HSF2BP recruitment

A recent study has reported that HSF2BP interacts with BRCA2 to mediate its localization to recombination sites. As with *Meiok21^−^^/^*^−^ spermatocytes, *Hsf2bp^−^^/^^−^* spermatocytes also have a normal level of DSBs and a low number of DMC1 and RAD51 foci (30). We further found that MEIOK21 interacted and colocalized with HSF2BP at recombination sites during meiosis. These results suggest that MEIOK21 may regulate DMC1/RAD51 foci through interacting with and modulating HSF2BP. Therefore, the chromosome localization of HSF2BP was examined in WT and *Meiok21^−^^/^^−^* spermatocytes. As reported, HSF2BP foci appeared at leptotene, reached the peak at zygotene, then gradually decreased and finally disappeared at late pachytene in WT (Figure 5K, L). However, only a low number of HSF2BP foci were detected in *Meiok21^−^^/^^−^* spermatocytes compared to WT at corresponding stages (e.g. 63.25 ± 3.229 versus 167.8 ± 6.786 at zygotene; Figure 5K, L). Thus, knockout of *Meiok21* leads to a significantly decreased number of HSF2BP foci on meiotic chromosomes.

## DISCUSSION

We identified a new protein, MEIOK21, that is preferentially expressed in testes and fetal ovaries. MEIOK21 specifically localizes on meiotic recombination sites in a DSB dependent manner, and colocalizes with recombination factors RPA and HSF2BP. The dynamic and unique localization patterns of MEIOK21 show that it is a meiotic recombination bridge. The C-terminal domain of MEIOK21 physically interacts with the N-terminal domain of HSF2BP. In *Meiok21^−^^/^^−^* spermatocytes, HSF2BP and the recombinases DMC1 and RAD51 cannot efficiently localize to recombination sites, which finally results in severe defects in synapsis and crossover recombination.

### MEIOK21 regulates DMC1/RAD51 foci through interaction with HSF2BP

The formation of MEIOK21 foci on meiotic chromosomes required SPO11 but was independent of DMC1. This observation suggests that MEIOK21 works after DSB formation but before DMC1. Further studies showed that *Meiok21* knockout does not affect RPA recruitment but the number of RAD51/DMC1 foci is significantly reduced. Thus, MEIOK21 probably works after DSB formation and RPA recruitment to regulate RAD51/DMC1 nucleoprotein filament assembly or their stability on chromosomes.

Efficient loading and stabilization of DMC1/RAD51 is required for proper homolog recombination. In *Dmc1* knockout mice, spermatocytes arrest at zygotene with severe defects in meiotic recombination and synapsis (44,54). The proper function of DMC1 and RAD51 requires many accessory proteins, which regulate the assembly of RAD51/DMC1 on ssDNA and/or the stability of the nucleoprotein filaments (20,22,25–27,30,69,70). Compared with core recombinase DMC1/RAD51, the absence of different accessory factors differentially impairs meiotic recombination and synapsis. The absence of some accessory factors, such as HOP2-MND1 and RPA, leads to rarely detectable RAD51/DMC1 or MLH1 foci and severe synapsis defect (29,71). However, the absence of other accessory factors causes only moderate meiotic defects. For example, in SWS1 or SWSAP1 knockout mice, although significantly decreased, the numbers of RAD51/DMC1 and MLH1 foci are still observed at a considerable level in both spermatocytes and oocytes (14,24). It is unclear how these accessory proteins work and collaborate to finely regulate meiotic recombination, and why so many factors are required.

MEIOK21 specifically interacts with HSF2BP (Figure 3 and Supplementary Figure S2). In *Meiok21^−^^/^^−^* spermatocytes, RAD51/DMC1 foci are detected at ∼1/3 of the WT level, and MLH1 foci are detected at ∼2/3 of WT level (Figure 5). These results suggest that MEIOK21 probably regulates RAD51/DMC1 foci formation and/or their stability likely through modulating HSF2BP. This proposal is supported by the fact that the number of HSF2BP foci dropped significantly in *Meiok21^−^^/^^−^* spermatocytes (Figure 5K, L). However, we cannot exclude the following possibilities: (i) MEIOK21 and HSF2BP regulate each other or cooperate for efficient formation of RAD51/DMC1 foci; (ii) MEIOK21 may directly regulate RAD51/DMC1 foci formation. However, the latter is less likely since no direct interaction is detected between MEIOK21 and RAD51 or DMC1 in our study (Supplementary Figure S2A).

Early studies showed that BRCA2 interacts with DMC1 and RAD51 (72–74). A recent study found that HSF2BP can directly interact with RPA and BRCA2, and the localization of GFP tagged BRCA2 is altered when it is electroporated into *Hsf2bp^−^^/^^−^* testes (30). Based on these results, the authors proposed that RPA recruits HSF2BP, which further recruits BRCA2-DMC1/RAD51 to DSB sites by direct physical interactions (30). However, this electroporation result may not reflect the *in vivo* functional interaction between BRCA2 and HSF2BP. Since BRCA2 is essential for both male and female meiosis (22), if HSF2BP is required to recruit BRCA2, *Hsf2bp* deficiency should severely affect both male and female meiosis. However, knockout of *Hsf2bp* only mildly affects female meiosis (22,28,30). Our results show that *Meiok21* knockout significantly decreases HSF2BP focus number but has little or no effect on BRCA2 (Supplementary Figure S7EF), and as with *Hsf2bp^−^^/^^−^* mutants, *Meiok21^−^^/^^−^* mutants severely impair male but not female meiosis. However, we cannot completely exclude the possibility that the residual low level of HSF2BP in *Meiok21* knockout spermatocytes is sufficient to recruit BRCA2 to recombination sites. Thus, further studies are required to elucidate the physical and functional interactions between HSF2BP and BRCA2.

### Meiotic homologous recombination bridges

Meiotic chromosomes are proposed to be organized as loop-axis structures (32,75). In this model, each chromatid is assembled in arrays of loops. The base of these loops is the structural ‘axis’, which is decorated with a set of proteins. Meiotic recombination occurs in the context of and is tightly regulated by this chromosome architecture (15,32,75). Pre-DSB complexes are loaded onto chromosome loops and then are tethered to the axis where DSBs are formed (32,76). Based on studies mainly from yeast, it has been proposed that after DSB ends are processed, the first ssDNA end is released from the chromosome axis to search and invade the homologous chromosome to form a D-loop (15,31,32,77). Thus, this process brings the homologous chromosomes close together. Subsequently, a small subset of these inter-homolog recombination intermediates is selected to become COs, while the rest become NCOs (4,15). Proteins involved in different recombination steps can be visualized by their different association status with chromosomes (78).

MEIOK21 first appears as foci on the chromosome axis at leptotene. During early zygotene, MEIOK21 foci are then released but still associated with the axis as ‘hanging foci’. Along with meiosis progression, homolog axes align closely and MEIOK21 ‘bridges’ appear to tightly link the two axes. Finally, when homologs synapsed, bigger and brighter single MEIOK21 foci, assumed to be fused foci, can be visualized between the two axes. These dynamic localization patterns are unique to meiotic recombination factors, such as RPA, RAD51, DMC1 and MSH4, in diverse species (31,32,45,46,77,79). This process has been well documented in *Sordaria* meiosis, where chromosomes in a single nucleus undergo meiosis synchronously and fully aligned homolog axes can be easily observed shortly before synapsis (31,45). However, in other organisms including mouse, different chromosomes progress asynchronously in the nucleus during meiosis (80). Moreover, different regions of a pair of homologs can be at significantly different stages, e.g. some regions of chromosome are synapsed, some regions are unsynapsed but homolog axes stay close, and some other regions of homolog axes stay far apart (Figure 1BD) (81). Recombination bridges only can be seen in the right homolog regions at the right time. Recombination bridges are composed of DNA, axis proteins, recombination complex, and mediator complex with intrinsic axis affinity independent of recombination, e.g. ZIP2–ZIP4 complex (31,32).

The DSB-dependent localization dynamics of MEIOK21 suggests that MEIOK21 is involved in recombination bridges as a member of the recombination complex. In the absence of MEIOK21, the numbers of DMC1 and RAD51 foci are greatly decreased. Therefore, MEIOK21 is required for efficient formation of meiotic recombination bridges. This hypothesis is consistent with the fact that CO recombination as visualized by MLH1 foci occurs but at a significantly decreased level in the absence of MEIOK21 (Figure 5CD). The decreased number of inter-homolog recombination cause defects in not only CO recombination but also synapsis as observed, because more homologous recombination interaction sites are required for efficient synapsis than for CO formation (68,82).

### The sexually dimorphic phenotype of *Meiok21* knockout mice

*Meiok21* is highly expressed in both mouse testes and fetal ovaries, but knockout of *Meiok21* showed sexually dimorphic phenotypes: males are infertile, but females have no obvious fertility defects (Supplementary Figure S4). Several other recombination mutants also showed sexual dimorphisms. Known recombination factors regulating DMC1/RAD51 can be classified into two groups. The first group includes HOP2-MND1, BRCA2, MEIOB and the Shu complex SWS1-SWSAP1, whose deletion or mutation leads to severe meiosis defects in both males and females (22,24,26,27,29,83). The second group includes ATR, HSF2BP, TEX15 and BRCA1. Deletion or mutation of these genes only affect male meiosis and fertility, but have no or little effects on female meiosis or fertility (20,25,28,30,69,70). MEIOK21 belongs to the second group. The existence of two different types of DMC1/RAD51 regulators suggests different mechanisms regulating meiotic recombination between males and females. One possibility is that the checkpoint is less stringent in females than that in males, thus meiotic errors could be tolerated in oogenesis but not in spermatogenesis (1,84,85). The consequence of the weak checkpoint in female meiosis is that oogenesis is error prone (86). Another possibility is that the functions of the second group of genes are male-specific, though some of them are also expressed during female meiosis, or their functions might be redundant in females.

## Supplementary Material

gkaa406_Supplemental_File

## References

[1] Nagaoka S.I., Hassold T.J., Hunt P.A. Human aneuploidy: mechanisms and new insights into an age-old problem. Nat. Rev. Genet. 2012; 13:493–504. 22705668 10.1038/nrg3245 PMC3551553

[2] Capalbo A., Hoffmann E.R., Cimadomo D., Ubaldi F.M., Rienzi L. Human female meiosis revised: new insights into the mechanisms of chromosome segregation and aneuploidies from advanced genomics and time-lapse imaging. Hum. Reprod. Update. 2017; 23:706–722. 28961822 10.1093/humupd/dmx026

[3] Wang S., Liu Y., Shang Y., Zhai B., Yang X., Kleckner N., Zhang L. Crossover interference, crossover maturation, and human aneuploidy. Bioessays. 2019; 41:e1800221. 31424607 10.1002/bies.201800221 PMC6756933

[4] Zickler D., Kleckner N. Recombination, pairing, and synapsis of homologs during meiosis. Cold Spring Harb. Perspect. Biol. 2015; 7:a016626. 25986558 10.1101/cshperspect.a016626 PMC4448610

[5] Keeney S., Giroux C.N., Kleckner N. Meiosis-specific DNA double-strand breaks are catalyzed by Spo11, a member of a widely conserved protein family. Cell. 1997; 88:375–384. 9039264 10.1016/s0092-8674(00)81876-0

[6] Romanienko P.J., Camerini-Otero R.D. The mouse Spo11 gene is required for meiotic chromosome synapsis. Mol. Cell. 2000; 6:975–987. 10.1016/s1097-2765(00)00097-6 11106738

[7] Baudat F., Manova K., Yuen J.P., Jasin M., Keeney S. Chromosome synapsis defects and sexually dimorphic meiotic progression in mice lacking Spo11. Mol. Cell. 2000; 6:989–998. 11106739 10.1016/s1097-2765(00)00098-8

[8] Bellani M.A., Boateng K.A., McLeod D., Camerini-Otero R.D. The expression profile of the major mouse SPO11 isoforms indicates that SPO11beta introduces double strand breaks and suggests that SPO11alpha has an additional role in prophase in both spermatocytes and oocytes. Mol. Cell. Biol. 2010; 30:4391–4403. 20647542 10.1128/MCB.00002-10 PMC2937527

[9] Lange J., Yamada S., Tischfield S.E., Pan J., Kim S., Zhu X., Socci N.D., Jasin M., Keeney S. The landscape of mouse meiotic double-strand break formation, processing, and repair. Cell. 2016; 167:695–708. 27745971 10.1016/j.cell.2016.09.035 PMC5117687

[10] Robert T., Nore A., Brun C., Maffre C., Crimi B., Bourbon H.M., de Massy B. The TopoVIB-Like protein family is required for meiotic DNA double-strand break formation. Science. 2016; 351:943–949. 26917764 10.1126/science.aad5309

[11] Lichten M., de Massy B. The impressionistic landscape of meiotic recombination. Cell. 2011; 147:267–270. 22000007 10.1016/j.cell.2011.09.038 PMC3263351

[12] Gray S., Cohen P.E. Control of meiotic crossovers: from double-strand break formation to designation. Annu. Rev. Genet. 2016; 50:175–210. 27648641 10.1146/annurev-genet-120215-035111 PMC5319444

[13] Brown M.S., Bishop D.K. DNA strand exchange and RecA homologs in meiosis. Cold Spring Harb. Perspect. Biol. 2014; 7:a016659. 25475089 10.1101/cshperspect.a016659 PMC4292170

[14] Matsuzaki K., Kondo S., Ishikawa T., Shinohara A. Human RAD51 paralogue SWSAP1 fosters RAD51 filament by regulating the anti-recombinase FIGNL1 AAA+ ATPase. Nat. Commun. 2019; 10:1407. 30926776 10.1038/s41467-019-09190-1 PMC6440994

[15] Hunter N. Meiotic recombination: the essence of heredity. Cold Spring Harb. Perspect. Biol. 2015; 7:a016618. 26511629 10.1101/cshperspect.a016618 PMC4665078

[16] Lynn A., Soucek R., Borner G.V. ZMM proteins during meiosis: crossover artists at work. Chromosome Res. 2007; 15:591–605. 17674148 10.1007/s10577-007-1150-1

[17] Pyatnitskaya A., Borde V., De Muyt A. Crossing and zipping: molecular duties of the ZMM proteins in meiosis. Chromosoma. 2019; 128:181–198. 31236671 10.1007/s00412-019-00714-8

[18] Wang S., Hassold T., Hunt P., White M.A., Zickler D., Kleckner N., Zhang L. Inefficient crossover maturation underlies elevated aneuploidy in human female meiosis. Cell. 2017; 168:977–989. 28262352 10.1016/j.cell.2017.02.002 PMC5408880

[19] Cloud V., Chan Y.L., Grubb J., Budke B., Bishop D.K. Rad51 is an accessory factor for Dmc1-mediated joint molecule formation during meiosis. Science. 2012; 337:1222–1225. 22955832 10.1126/science.1219379 PMC4056682

[20] Yang F., Eckardt S., Leu N.A., McLaughlin K.J., Wang P.J. Mouse TEX15 is essential for DNA double-strand break repair and chromosomal synapsis during male meiosis. J. Cell Biol. 2008; 180:673–679. 18283110 10.1083/jcb.200709057 PMC2265566

[21] Gudmundsdottir K., Ashworth A. BRCA2 in meiosis: turning over a new leaf. Trends Cell Biol. 2004; 14:401–404. 15308204 10.1016/j.tcb.2004.07.002

[22] Sharan S.K., Pyle A., Coppola V., Babus J., Swaminathan S., Benedict J., Swing D., Martin B.K., Tessarollo L., Evans J.P. et al. BRCA2 deficiency in mice leads to meiotic impairment and infertility. Development. 2004; 131:131–142. 14660434 10.1242/dev.00888

[23] Zhang F., Ma J., Wu J., Ye L., Cai H., Xia B., Yu X. PALB2 links BRCA1 and BRCA2 in the DNA-damage response. Curr. Biol. 2009; 19:524–529. 19268590 10.1016/j.cub.2009.02.018 PMC2750839

[24] Abreu C.M., Prakash R., Romanienko P.J., Roig I., Keeney S., Jasin M. Shu complex SWS1-SWSAP1 promotes early steps in mouse meiotic recombination. Nat. Commun. 2018; 9:3961. 30305635 10.1038/s41467-018-06384-x PMC6180034

[25] Pacheco S., Maldonado-Linares A., Marcet-Ortega M., Rojas C., Martinez-Marchal A., Fuentes-Lazaro J., Lange J., Jasin M., Keeney S., Fernandez-Capetillo O. et al. ATR is required to complete meiotic recombination in mice. Nat. Commun. 2018; 9:2622. 29977027 10.1038/s41467-018-04851-z PMC6033890

[26] Souquet B., Abby E., Herve R., Finsterbusch F., Tourpin S., Le Bouffant R., Duquenne C., Messiaen S., Martini E., Bernardino-Sgherri J. et al. MEIOB targets single-strand DNA and is necessary for meiotic recombination. PLos Genet. 2013; 9:e1003784. 24068956 10.1371/journal.pgen.1003784 PMC3778009

[27] Luo M., Yang F., Leu N.A., Landaiche J., Handel M.A., Benavente R., La Salle S., Wang P.J. MEIOB exhibits single-stranded DNA-binding and exonuclease activities and is essential for meiotic recombination. Nat. Commun. 2013; 4:2788. 24240703 10.1038/ncomms3788 PMC3891831

[28] Brandsma I., Sato K., van Rossum-Fikkert S.E., van Vliet N., Sleddens E., Reuter M., Odijk H., van den Tempel N., Dekkers D.H.W., Bezstarosti K. et al. HSF2BP interacts with a conserved domain of BRCA2 and is required for mouse spermatogenesis. Cell Rep. 2019; 27:3790–3798. 31242413 10.1016/j.celrep.2019.05.096

[29] Pezza R.J., Voloshin O.N., Volodin A.A., Boateng K.A., Bellani M.A., Mazin A.V., Camerini-Otero R.D. The dual role of HOP2 in mammalian meiotic homologous recombination. Nucleic Acids Res. 2014; 42:2346–2357. 24304900 10.1093/nar/gkt1234 PMC3936763

[30] Zhang J., Fujiwara Y., Yamamoto S., Shibuya H. A meiosis-specific BRCA2 binding protein recruits recombinases to DNA double-strand breaks to ensure homologous recombination. Nat. Commun. 2019; 10:722. 30760716 10.1038/s41467-019-08676-2 PMC6374363

[31] Dubois E., De Muyt A., Soyer J.L., Budin K., Legras M., Piolot T., Debuchy R., Kleckner N., Zickler D., Espagne E. Building bridges to move recombination complexes. Proc. Natl Acad. Sci. USA. 2019; 116:12400–12409. 31147459 10.1073/pnas.1901237116 PMC6589682

[32] Zickler D., Kleckner N. Meiotic chromosomes: integrating structure and function. Annu. Rev. Genet. 1999; 33:603–754. 10690419 10.1146/annurev.genet.33.1.603

[33] Liu H., Huang T., Li M., Li M., Zhang C., Jiang J., Yu X., Yin Y., Zhang F., Lu G. et al. SCRE serves as a unique synaptonemal complex fastener and is essential for progression of meiosis prophase I in mice. Nucleic Acids Res. 2019; 47:5670–5683. 30949703 10.1093/nar/gkz226 PMC6582318

[34] Li M., Huang T., Li M.J., Zhang C.X., Yu X.C., Yin Y.Y., Liu C., Wang X., Feng H.W., Zhang T. et al. The histone modification reader ZCWPW1 is required for meiosis prophase I in male but not in female mice. Sci Adv. 2019; 5:eaax1101. 31453335 10.1126/sciadv.aax1101 PMC6693912

[35] Shang Y., Zhu F., Wang L., Ouyang Y.C., Dong M.Z., Liu C., Zhao H., Cui X., Ma D., Zhang Z. et al. Essential role for SUN5 in anchoring sperm head to the tail. Elife. 2017; 6:e28199. 28945193 10.7554/eLife.28199 PMC5634783

[36] Hess R.A., Renato de Franca L. Spermatogenesis and cycle of the seminiferous epithelium. Adv. Exp. Med. Biol. 2008; 636:1–15. 19856159 10.1007/978-0-387-09597-4_1

[37] Peters A.H., Plug A.W., van Vugt M.J., de Boer P. A drying-down technique for the spreading of mammalian meiocytes from the male and female germline. Chromosome Res. 1997; 5:66–68. 9088645 10.1023/a:1018445520117

[38] Yue F., Cheng Y., Breschi A., Vierstra J., Wu W., Ryba T., Sandstrom R., Ma Z., Davis C., Pope B.D. et al. A comparative encyclopedia of DNA elements in the mouse genome. Nature. 2014; 515:355–364. 25409824 10.1038/nature13992 PMC4266106

[39] Fagerberg L., Hallstrom B.M., Oksvold P., Kampf C., Djureinovic D., Odeberg J., Habuka M., Tahmasebpoor S., Danielsson A., Edlund K. et al. Analysis of the human tissue-specific expression by genome-wide integration of transcriptomics and antibody-based proteomics. Mol. Cell. Proteomics. 2014; 13:397–406. 24309898 10.1074/mcp.M113.035600 PMC3916642

[40] Reynolds A., Qiao H., Yang Y., Chen J.K., Jackson N., Biswas K., Holloway J.K., Baudat F., de Massy B., Wang J. et al. RNF212 is a dosage-sensitive regulator of crossing-over during mammalian meiosis. Nat. Genet. 2013; 45:269–278. 23396135 10.1038/ng.2541 PMC4245152

[41] Qiao H., Chen J.K., Reynolds A., Hoog C., Paddy M., Hunter N. Interplay between synaptonemal complex, homologous recombination, and centromeres during mammalian meiosis. PLos Genet. 2012; 8:e1002790. 22761591 10.1371/journal.pgen.1002790 PMC3386176

[42] Bhat K.P., Cortez D. RPA and RAD51: fork reversal, fork protection, and genome stability. Nat. Struct. Mol. Biol. 2018; 25:446–453. 29807999 10.1038/s41594-018-0075-z PMC6006513

[43] Dai J., Voloshin O., Potapova S., Camerini-Otero R.D. Meiotic knockdown and complementation reveals essential role of RAD51 in mouse spermatogenesis. Cell Rep. 2017; 18:1383–1394. 28178517 10.1016/j.celrep.2017.01.024 PMC5358547

[44] Pittman D.L., Cobb J., Schimenti K.J., Wilson L.A., Cooper D.M., Brignull E., Handel M.A., Schimenti J.C. Meiotic prophase arrest with failure of chromosome synapsis in mice deficient for Dmc1, a germline-specific RecA homolog. Mol. Cell. 1998; 1:697–705. 9660953 10.1016/s1097-2765(00)80069-6

[45] Storlazzi A., Gargano S., Ruprich-Robert G., Falque M., David M., Kleckner N., Zickler D. Recombination proteins mediate meiotic spatial chromosome organization and pairing. Cell. 2010; 141:94–106. 20371348 10.1016/j.cell.2010.02.041 PMC2851631

[46] Oliver-Bonet M., Campillo M., Turek P.J., Ko E., Martin R.H. Analysis of replication protein A (RPA) in human spermatogenesis. Mol. Hum. Reprod. 2007; 13:837–844. 17981954 10.1093/molehr/gam076

[47] Boateng K.A., Bellani M.A., Gregoretti I.V., Pratto F., Camerini-Otero R.D. Homologous pairing preceding SPO11-mediated double-strand breaks in mice. Dev. Cell. 2013; 24:196–205. 23318132 10.1016/j.devcel.2012.12.002 PMC3562373

[48] Bishop D.K., Park D., Xu L., Kleckner N. DMC1: a meiosis-specific yeast homolog of E. coli recA required for recombination, synaptonemal complex formation, and cell cycle progression. Cell. 1992; 69:439–456. 1581960 10.1016/0092-8674(92)90446-j

[49] Hays E., Majchrzak N., Daniel V., Ferguson Z., Brown S., Hathorne K., La Salle S. Spermatogenesis associated 22 is required for DNA repair and synapsis of homologous chromosomes in mouse germ cells. Andrology. 2017; 5:299–312. 28297563 10.1111/andr.12315 PMC5354093

[50] Ishishita S., Matsuda Y., Kitada K. Genetic evidence suggests that Spata22 is required for the maintenance of Rad51 foci in mammalian meiosis. Sci. Rep. 2014; 4:6148. 25142975 10.1038/srep06148 PMC4139951

[51] Wu Y., Liao S., Wang X., Wang S., Wang M., Han C. HSF2BP represses BNC1 transcriptional activity by sequestering BNC1 to the cytoplasm. FEBS Lett. 2013; 587:2099–2104. 23707421 10.1016/j.febslet.2013.04.049

[52] Yoshima T., Yura T., Yanagi H. Novel testis-specific protein that interacts with heat shock factor 2. Gene. 1998; 214:139–146. 9651507 10.1016/s0378-1119(98)00208-x

[53] Edelmann W., Cohen P.E., Kneitz B., Winand N., Lia M., Heyer J., Kolodner R., Pollard J.W., Kucherlapati R. Mammalian MutS homologue 5 is required for chromosome pairing in meiosis. Nat. Genet. 1999; 21:123–127. 9916805 10.1038/5075

[54] Yoshida K., Kondoh G., Matsuda Y., Habu T., Nishimune Y., Morita T. The mouse RecA-like gene Dmc1 is required for homologous chromosome synapsis during meiosis. Mol. Cell. 1998; 1:707–718. 9660954 10.1016/s1097-2765(00)80070-2

[55] Ahmed E.A., de Rooij D.G. Staging of mouse seminiferous tubule cross-sections. Methods Mol. Biol. 2009; 558:263–277. 19685330 10.1007/978-1-60761-103-5_16

[56] de Vries F.A., de Boer E., van den Bosch M., Baarends W.M., Ooms M., Yuan L., Liu J.G., van Zeeland A.A., Heyting C., Pastink A. Mouse Sycp1 functions in synaptonemal complex assembly, meiotic recombination, and XY body formation. Genes Dev. 2005; 19:1376–1389. 15937223 10.1101/gad.329705 PMC1142560

[57] Bannister L.A., Schimenti J.C. Homologous recombinational repair proteins in mouse meiosis. Cytogenet. Genome Res. 2004; 107:191–200. 15467364 10.1159/000080597

[58] Anderson L.K., Reeves A., Webb L.M., Ashley T. Distribution of crossing over on mouse synaptonemal complexes using immunofluorescent localization of MLH1 protein. Genetics. 1999; 151:1569–1579. 10101178 10.1093/genetics/151.4.1569 PMC1460565

[59] Marcon E., Moens P. MLH1p and MLH3p localize to precociously induced chiasmata of okadaic-acid-treated mouse spermatocytes. Genetics. 2003; 165:2283–2287. 14704203 10.1093/genetics/165.4.2283 PMC1462919

[60] Baker S.M., Plug A.W., Prolla T.A., Bronner C.E., Harris A.C., Yao X., Christie D.M., Monell C., Arnheim N., Bradley A. et al. Involvement of mouse Mlh1 in DNA mismatch repair and meiotic crossing over. Nat. Genet. 1996; 13:336–342. 8673133 10.1038/ng0796-336

[61] Cole F., Kauppi L., Lange J., Roig I., Wang R., Keeney S., Jasin M. Homeostatic control of recombination is implemented progressively in mouse meiosis. Nat. Cell Biol. 2012; 14:424–430. 22388890 10.1038/ncb2451 PMC3319518

[62] Mahadevaiah S.K., Turner J.M., Baudat F., Rogakou E.P., de Boer P., Blanco-Rodriguez J., Jasin M., Keeney S., Bonner W.M., Burgoyne P.S. Recombinational DNA double-strand breaks in mice precede synapsis. Nat. Genet. 2001; 27:271–276. 11242108 10.1038/85830

[63] Lam I., Keeney S. Mechanism and regulation of meiotic recombination initiation. Cold Spring Harb. Perspect. Biol. 2014; 7:a016634. 25324213 10.1101/cshperspect.a016634 PMC4292169

[64] Ribeiro J., Abby E., Livera G., Martini E. RPA homologs and ssDNA processing during meiotic recombination. Chromosoma. 2016; 125:265–276. 26520106 10.1007/s00412-015-0552-7 PMC4830875

[65] Lao J.P., Cloud V., Huang C.C., Grubb J., Thacker D., Lee C.Y., Dresser M.E., Hunter N., Bishop D.K. Meiotic crossover control by concerted action of Rad51-Dmc1 in homolog template bias and robust homeostatic regulation. PLos Genet. 2013; 9:e1003978. 24367271 10.1371/journal.pgen.1003978 PMC3868528

[66] Martini E., Diaz R.L., Hunter N., Keeney S. Crossover homeostasis in yeast meiosis. Cell. 2006; 126:285–295. 16873061 10.1016/j.cell.2006.05.044 PMC1949389

[67] Jones G.H., Franklin F.C. Meiotic crossing-over: obligation and interference. Cell. 2006; 126:246–248. 16873056 10.1016/j.cell.2006.07.010

[68] Zhang L., Espagne E., de Muyt A., Zickler D., Kleckner N.E. Interference-mediated synaptonemal complex formation with embedded crossover designation. Proc. Natl. Acad. Sci. U.S.A. 2014; 111:E5059–E5068. 25380597 10.1073/pnas.1416411111 PMC4250137

[69] Widger A., Mahadevaiah S.K., Lange J., ElInati E., Zohren J., Hirota T., Pacheco S., Maldonado-Linares A., Stanzione M., Ojarikre O. et al. ATR is a multifunctional regulator of male mouse meiosis. Nat. Commun. 2018; 9:2621. 29976923 10.1038/s41467-018-04850-0 PMC6033951

[70] Xu X., Aprelikova O., Moens P., Deng C.X., Furth P.A. Impaired meiotic DNA-damage repair and lack of crossing-over during spermatogenesis in BRCA1 full-length isoform deficient mice. Development. 2003; 130:2001–2012. 12642502 10.1242/dev.00410

[71] Shi B., Xue J., Yin H., Guo R., Luo M., Ye L., Shi Q., Huang X., Liu M., Sha J. et al. Dual functions for the ssDNA-binding protein RPA in meiotic recombination. PLos Genet. 2019; 15:e1007952. 30716097 10.1371/journal.pgen.1007952 PMC6375638

[72] Davies A.A., Masson J.Y., McIlwraith M.J., Stasiak A.Z., Stasiak A., Venkitaraman A.R., West S.C. Role of BRCA2 in control of the RAD51 recombination and DNA repair protein. Mol. Cell. 2001; 7:273–282. 11239456 10.1016/s1097-2765(01)00175-7

[73] Martinez J.S., von Nicolai C., Kim T., Ehlen A., Mazin A.V., Kowalczykowski S.C., Carreira A. BRCA2 regulates DMC1-mediated recombination through the BRC repeats. Proc. Natl Acad. Sci. U.S.A. 2016; 113:3515–3520. 26976601 10.1073/pnas.1601691113 PMC4822569

[74] Thorslund T., Esashi F., West S.C. Interactions between human BRCA2 protein and the meiosis-specific recombinase DMC1. EMBO J. 2007; 26:2915–2922. 17541404 10.1038/sj.emboj.7601739 PMC1894777

[75] Kleckner N. Chiasma formation: chromatin/axis interplay and the role(s) of the synaptonemal complex. Chromosoma. 2006; 115:175–194. 16555016 10.1007/s00412-006-0055-7

[76] Panizza S., Mendoza M.A., Berlinger M., Huang L., Nicolas A., Shirahige K., Klein F. Spo11-accessory proteins link double-strand break sites to the chromosome axis in early meiotic recombination. Cell. 2011; 146:372–383. 21816273 10.1016/j.cell.2011.07.003

[77] Tarsounas M., Morita T., Pearlman R.E., Moens P.B. RAD51 and DMC1 form mixed complexes associated with mouse meiotic chromosome cores and synaptonemal complexes. J. Cell Biol. 1999; 147:207–220. 10525529 10.1083/jcb.147.2.207 PMC2174216

[78] Zickler D., Kleckner N. A few of our favorite things: Pairing, the bouquet, crossover interference and evolution of meiosis. Semin. Cell Dev. Biol. 2016; 54:135–148. 26927691 10.1016/j.semcdb.2016.02.024 PMC4867269

[79] Moens P.B., Marcon E., Shore J.S., Kochakpour N., Spyropoulos B. Initiation and resolution of interhomolog connections: crossover and non-crossover sites along mouse synaptonemal complexes. J. Cell Sci. 2007; 120:1017–1027. 17344431 10.1242/jcs.03394

[80] Scherthan H., Schonborn I. Asynchronous chromosome pairing in male meiosis of the rat (Rattus norvegicus). Chromosome Res. 2001; 9:273–282. 11419792 10.1023/a:1016642528981

[81] Zickler D., Kleckner N. The leptotene-zygotene transition of meiosis. Annu. Rev. Genet. 1998; 32:619–697. 9928494 10.1146/annurev.genet.32.1.619

[82] Henderson K.A., Keeney S. Tying synaptonemal complex initiation to the formation and programmed repair of DNA double-strand breaks. Proc. Natl Acad. Sci. U.S.A. 2004; 101:4519–4524. 15070750 10.1073/pnas.0400843101 PMC384779

[83] Weinberg-Shukron A., Rachmiel M., Renbaum P., Gulsuner S., Walsh T., Lobel O., Dreifuss A., Ben-Moshe A., Zeligson S., Segel R. et al. Essential role of BRCA2 in ovarian development and function. N. Engl. J. Med. 2018; 379:1042–1049. 30207912 10.1056/NEJMoa1800024 PMC6230262

[84] Morelli M.A., Cohen P.E. Not all germ cells are created equal: aspects of sexual dimorphism in mammalian meiosis. Reproduction. 2005; 130:761–781. 16322537 10.1530/rep.1.00865

[85] Hunt P.A., Hassold T.J. Sex matters in meiosis. Science. 2002; 296:2181–2183. 12077403 10.1126/science.1071907

[86] El Yakoubi W., Wassmann K. Meiotic divisions: no place for gender equality. Adv. Exp. Med. Biol. 2017; 1002:1–17. 28600780 10.1007/978-3-319-57127-0_1

