# Supplementary material for: MEIOK21: a new component of meiotic recombination bridges required for spermatogenesis
Source: Nucleic Acids Res. 2020 May 28;48(12):6624–39. doi: 10.1093/nar/gkaa406 (PMC7337969; doi:10.1093/nar/gkaa406)
Supplement: gkaa406_Supplemental_File [file gkaa406_supplemental_file.pdf]

A

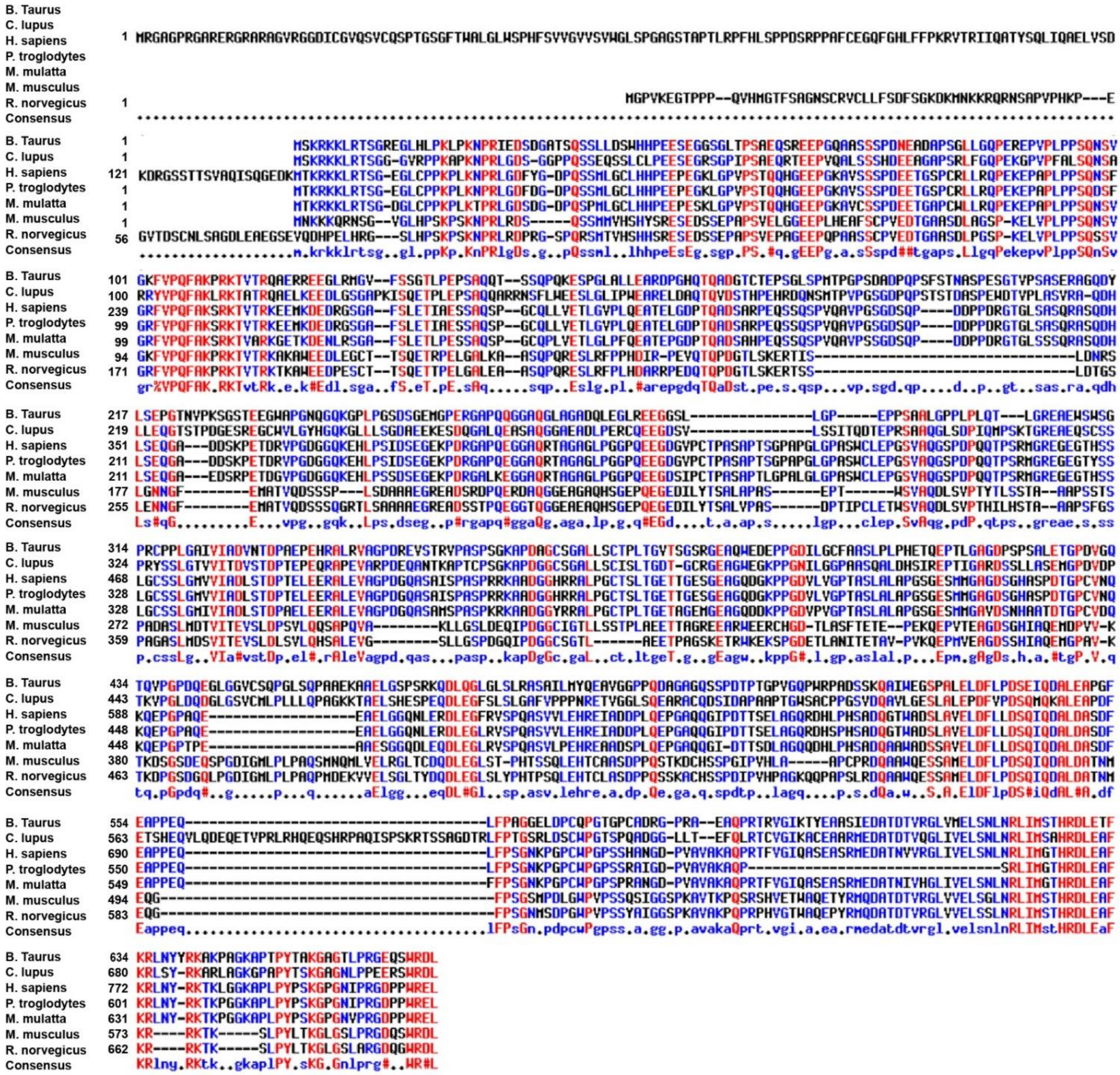

B

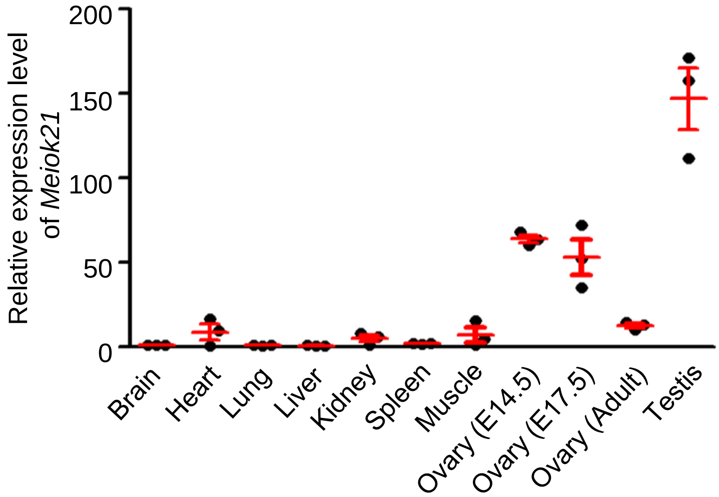

C

Tissue	ΔΔCT
Brain	0
Heart	-1.92±2.46
Lung	0.25±0.34
Liver	0.41±0.60
Kidney	-1.98±1.15
Spleen	-0.96±0.11
Muscle	-2.17±1.48
Ovary (E14.5)	-5.99±0.07
Ovary (E17.5)	-5.67±0.42
Ovary (Adult)	-3.63±0.24
Testis	-7.17±0.27

Supplementary Figure S1. MEIOK21 is highly conserved in vertebrates and is highly expressed in testis and fetal ovary. **(A)** MEIOK21 protein sequences from different species were obtained from the NCBI protein database, and the alignment was performed using Multalin (http://multalin.toulouse.inra.fr/multalin/). (**B**) *Meiok21* gene expression in different mouse tissues was examined by RT-qPCR (n=3). Error bar, mean ± SEM. **(C)** The ΔΔCT values (mean ± SEM) of RT-qPCR in (B); the data were normalized with expression level of *Meiok21* in brain.

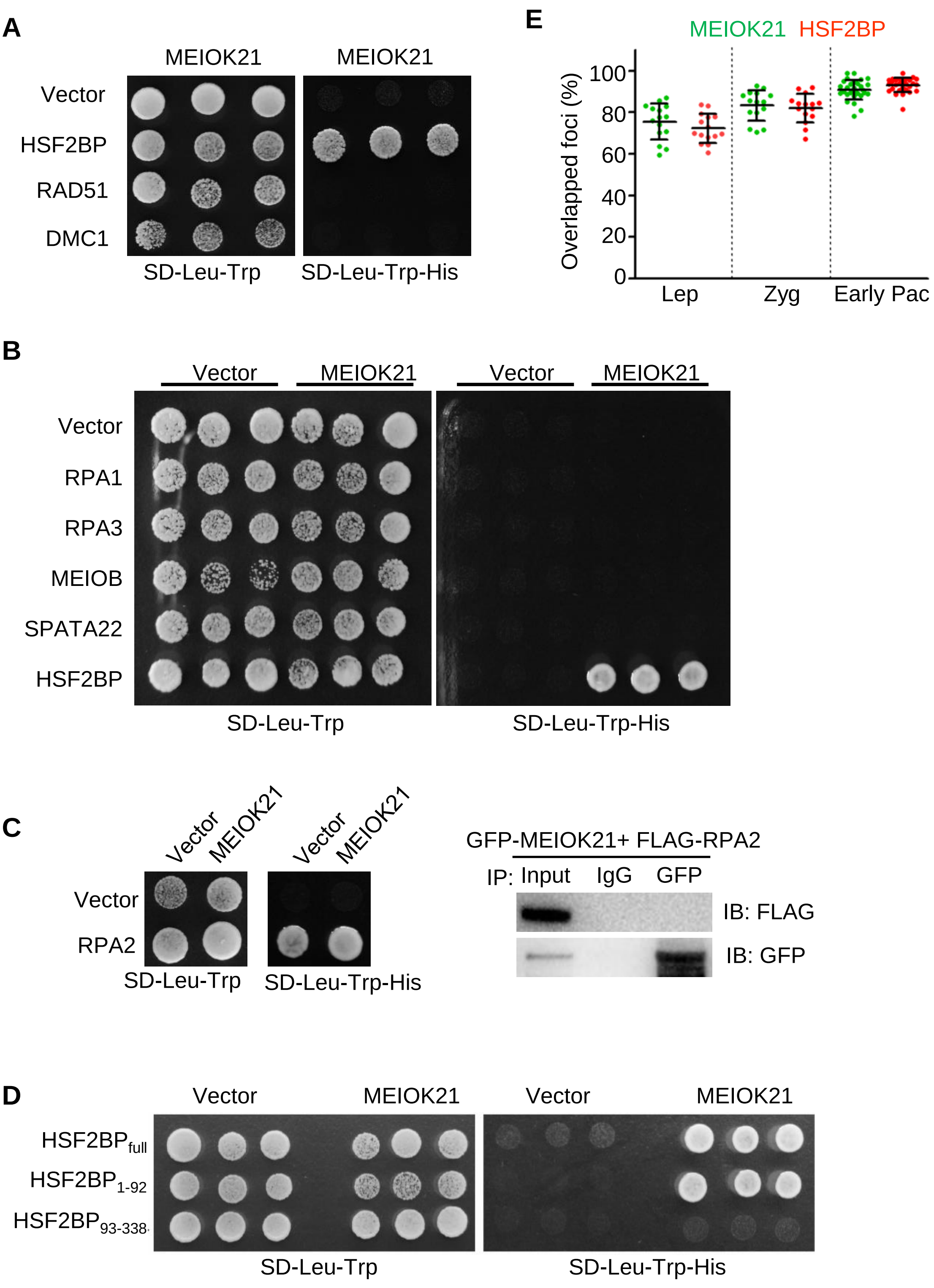

Supplementary Figure S2. Y2H screening of MEIOK21 interactors and interacting domains. In Y2H screening, when MEIOK21 was used as the bait, it showed self-activation. Therefore, MEIOK21 was used as the prey. **(A)** Y2H screening for interactions between MEIOK21 and RAD51, DMC1 or HSF2BP. **(B)** Y2H screening for interactions between MEIOK21 and RPA1, RPA3, MEIOB, SPATA22 or HSF2BP. (**C**) RPA2-BD shows self-activation (left). No interaction was detected between MEIOK21 and RPA2 by co-IP (right). (**D**) N-terminal domain (a.a. 1-92) of HSF2BP interacts with MEIOK21 as efficiently as the full length. All experiments were repeated three times. (**E**) Colocalization between MEIOK21 and HSF2BP. The number of spermatocytes from left to right, n=15, 15, 15, 15, 30 and 30, respectively. Error bar, mean ± SD.

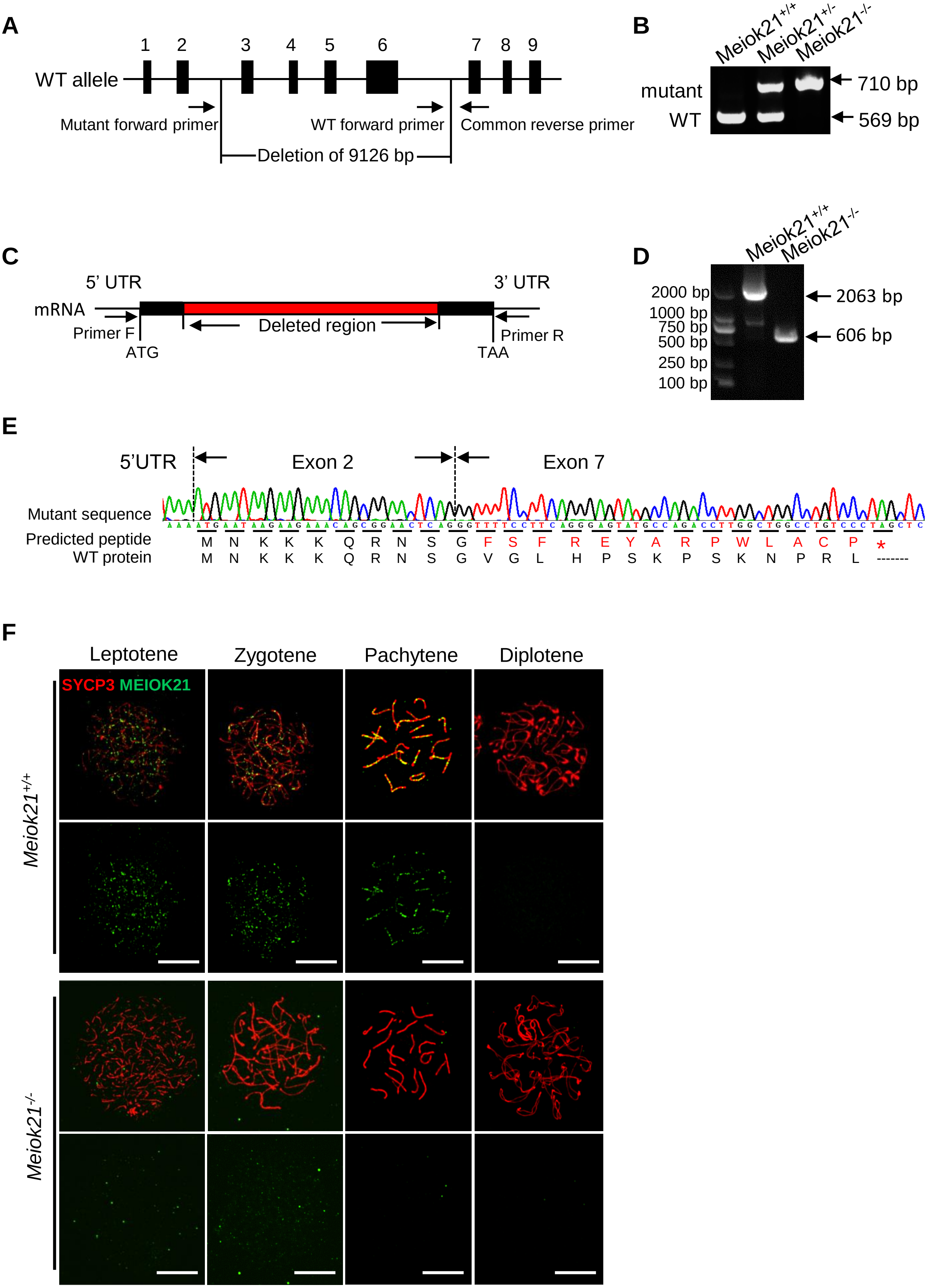

A
1 2 3 4 5 6 7 8 9
WT allele
Mutant forward primer
WT forward primer
Common reverse primer
Deletion of 9126 bp
B
Meiok21+/+
Meiok21+/-
Meiok21-/-
mutant
WT
710 bp
569 bp
C
5' UTR
3' UTR
mRNA
Primer F
Deleted region
Primer R
ATG
TAA
D
Meiok21+/+
Meiok21-/-
2000 bp
1000 bp
750 bp
500 bp
250 bp
100 bp
2063 bp
606 bp
E
5'UTR
Exon 2
Exon 7
Mutant sequence
Predicted peptide
WT protein
M N K K K Q R N S G F S F R E Y A R P W L A C P *
M N K K K Q R N S G V G L H P S K P S K N P R L -------
F
Leptotene
Zygotene
Pachytene
Diplotene
SYCP3 MEIOK21
Meiok21+/+
Meiok21-/-

Supplementary Figure S3. The schematics and validation of *Meiok21* gene knockout. (**A**) The *Meiok21* gene knockout strategy, a fragment of 9,126 bp harboring the exons 3-6 was deleted in the mutant. The positions of primers for genotyping are indicated. (**B**) The representative image of PCR genotyping in WT, *Meiok21*$^{+/-}$ and *Meiok21*$^{-/-}$ mice. (**C**) A cartoon shows the positions of primers used for RT-PCR. (**D**) As expected, a long transcript (~2,000 bp) was detected from WT testis and a short transcript (~600 bp) was detected in mutant testis by RT-PCR with primers described in (C). (**E**) Sequencing result of the RT-PCR product in (D) from mutant testis. If this short transcript was translated, it would be a short peptide of 24 amino acids (with frameshift from 11th amino acid residue) sharing only the first ten amino acids with WT MEIOK21. (**F**) Abundant MEIOK21 foci existed in WT spermatocytes from leptotene to pachytene, but no MEIOK21 signal was observed in *Meiok21*$^{-/-}$ spermatocytes. Scale bar, 10 μm.

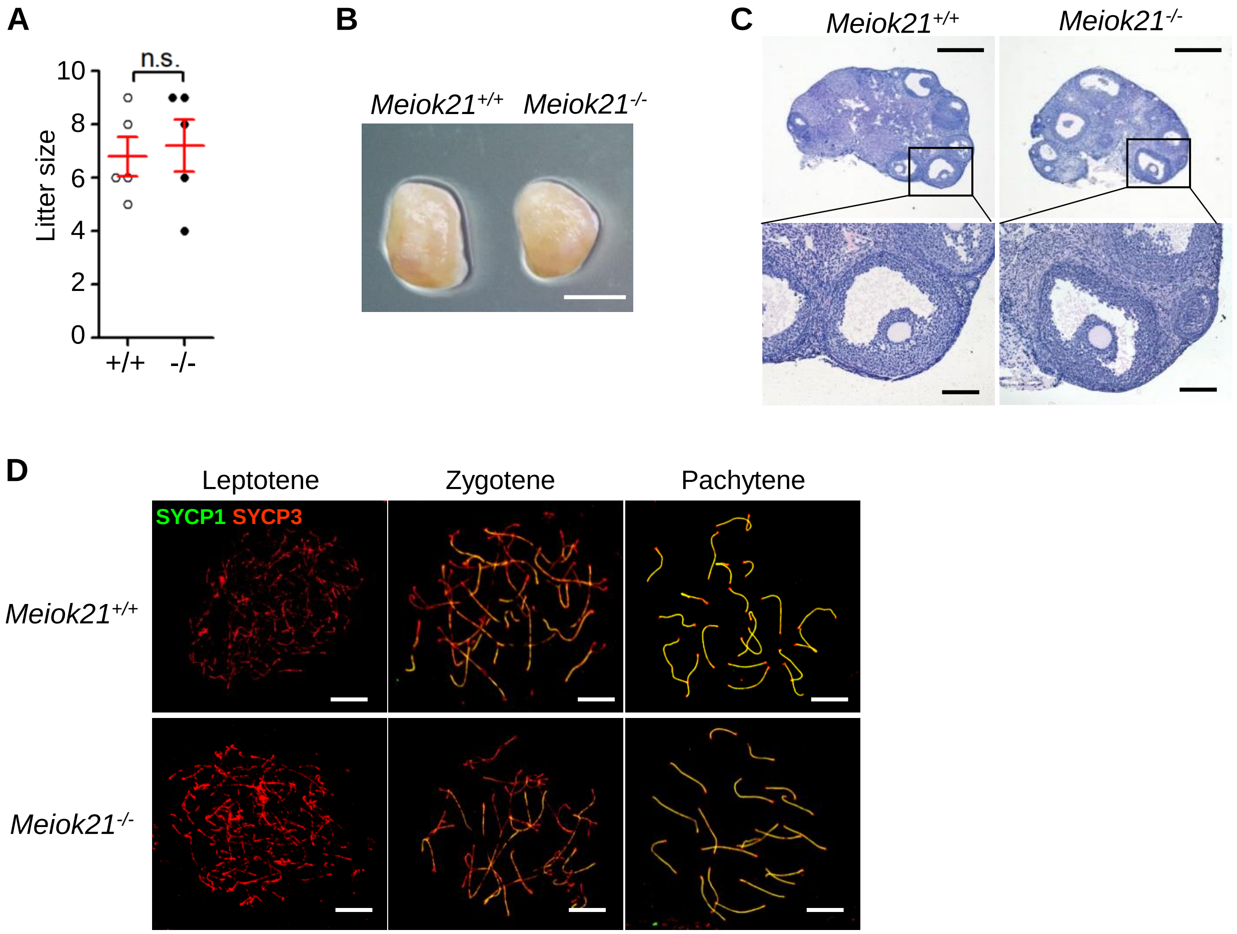

Supplementary Figure S4. MEIOK21 deficiency has no obvious influence on female fertility. (A) Litter size of WT and *Meiok21*$^{-/-}$ female mice (five female mice for each genotype). Error bar, mean ± SEM. **(B)** Adult ovary of WT and *Meiok21*$^{-/-}$ mice. Scale bar, 1 mm **(C)** HE staining of adult ovary from WT and *Meiok21*$^{-/-}$ mice. Scale bar, 200 μm (upper two panels); 20 μm (lower two panels). **(D)** No obvious synapsis defect was observed in E14.5 *Meiok21*$^{-/-}$ oocytes by immunostaining of SYCP3 (red) and SYCP1 (green). Scale bar, 10 μm.

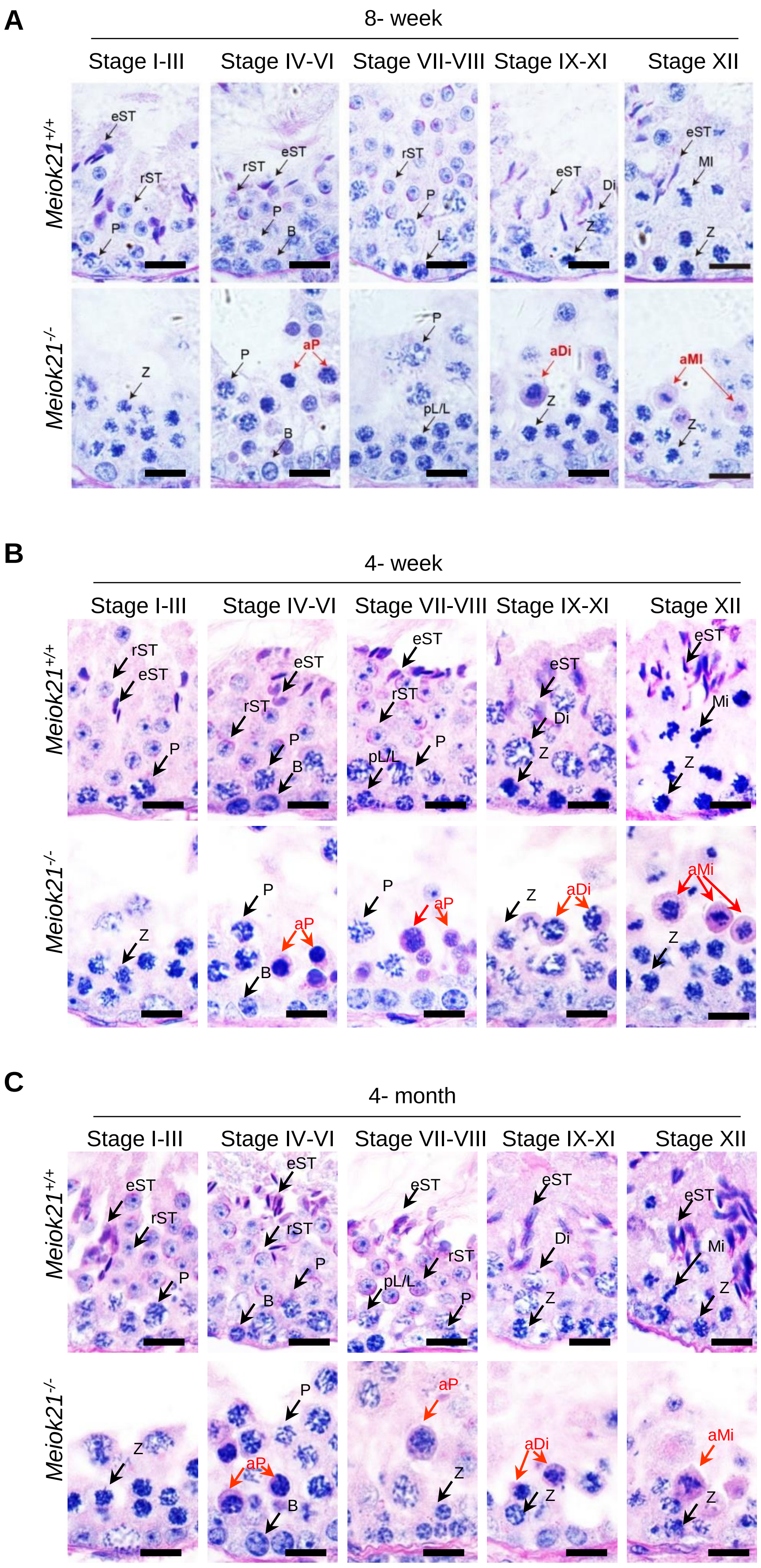
A
8- week
Stage I-III
Stage IV-VI
Stage VII-VIII
Stage IX-XI
Stage XII
Meiok21+/+
Meiok21-/-
eST
rST
P
B
L
Z
Di
MI
aP
pL/L
aDi
aMI
B
4- week
Stage I-III
Stage IV-VI
Stage VII-VIII
Stage IX-XI
Stage XII
Meiok21+/+
Meiok21-/-
rST
eST
P
B
pL/L
Di
Z
Mi
aP
aDi
aMi
C
4- month
Stage I-III
Stage IV-VI
Stage VII-VIII
Stage IX-XI
Stage XII
Meiok21+/+
Meiok21-/-
eST
rST
P
B
pL/L
Di
Z
Mi
aP
aDi
aMi

Supplementary Figure S5. MEIOK21 is required for normal meiosis. (A) Periodic acid Schiff (PAS) staining was performed for testis sections from 8-week-old WT and *Meiok21*$^{-/-}$ mice. Seminiferous tubules from stage I to stage XII could be observed in both WT and *Meiok21*$^{-/-}$ testis, but no spermatids were found in any seminiferous tubules in *Meiok21*$^{-/-}$ mice. Apoptotic spermatocytes appeared in various developmental stages including pachytene, diplotene and metaphase I (indicated by aP, aDi and aMI, respectively). Note, seminiferous tubule stages in *Meiok21*$^{-/-}$ mice were judged by the presence of spermatogonia and spermatocytes. Scale bar, 10 μm. PAS staining was performed for testis sections from 4-week-old **(B)** and 4-month-old **(C)** WT and *Meiok21*$^{-/-}$ mice.

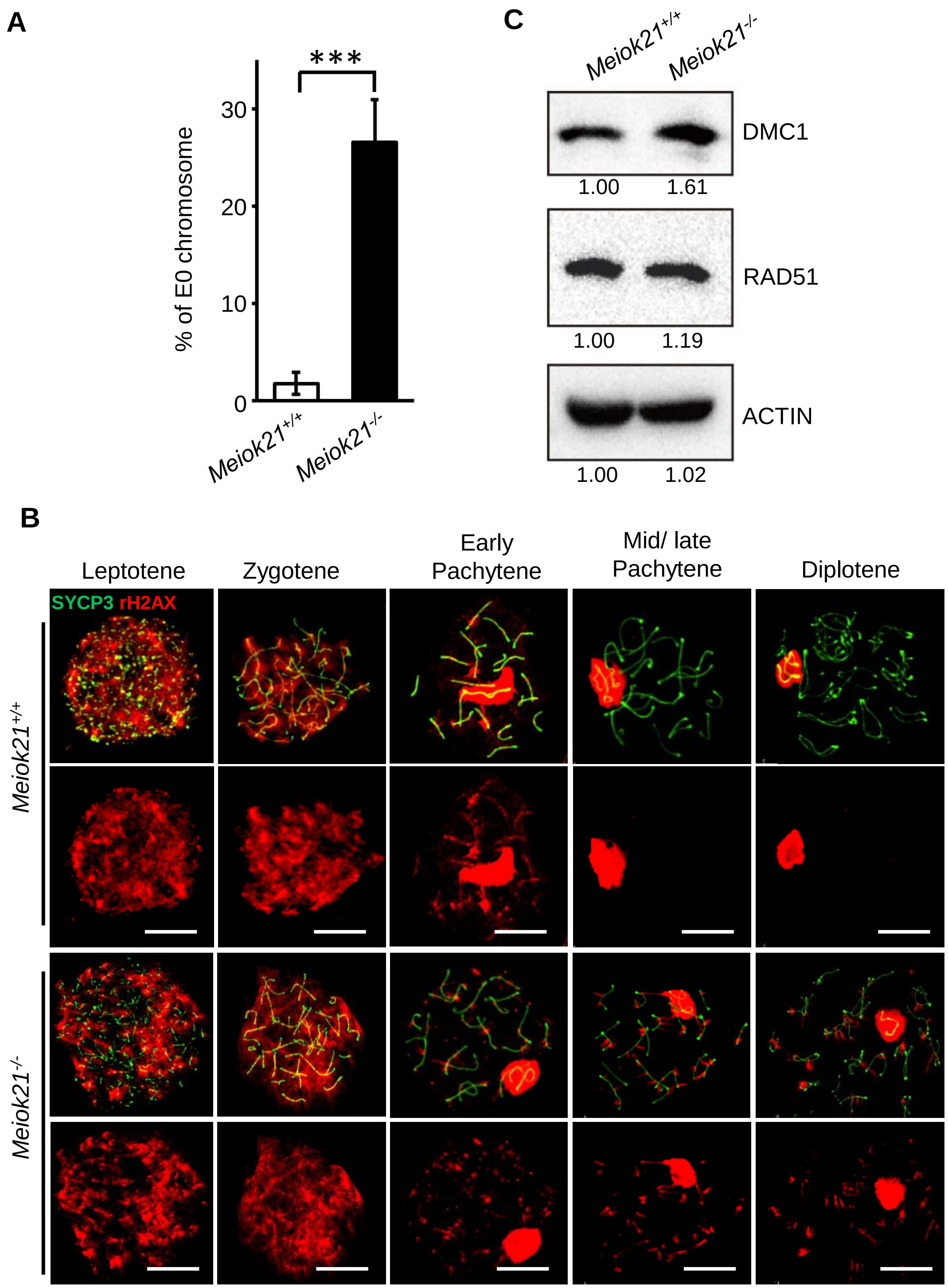

Supplementary Figure S6. Ablation of MEIOK21 impairs DSB repair and crossover recombination. (A) The percentage of pachytene chromosomes with no MLH1 foci (E0 chromosomes) is significantly elevated in *Meiok21*$^{-/-}$ (WT, n=551; *Meiok21*$^{-/-}$, n=399). Error bar, 95% confidence interval (CI), which is calculated according to CI = p $\pm$ 1.96*sqrt (p*(1-p)/n). **(B)** Comparable γH2AX can be observed in WT and *Meiok21*$^{-/-}$ spermatocytes at leptotene and zygotene. In WT spermatocytes, γH2AX signals on autosomes decreased at early pachytene and disappeared at mid/late pachytene. In *Meiok21*$^{-/-}$ spermatocytes, γH2AX signals on autosomes also decreased at early pachytene, but a number of γH2AX signals were still observed at mid-late pachytene and diplotene. Scale bar, 10 μm. **(C)** Slightly increased levels of RAD51 and DMC1 proteins in *Meiok21*$^{-/-}$ testes as revealed by Western blotting. The intensities of bands were quantified by Image J. The experiment was repeated twice.

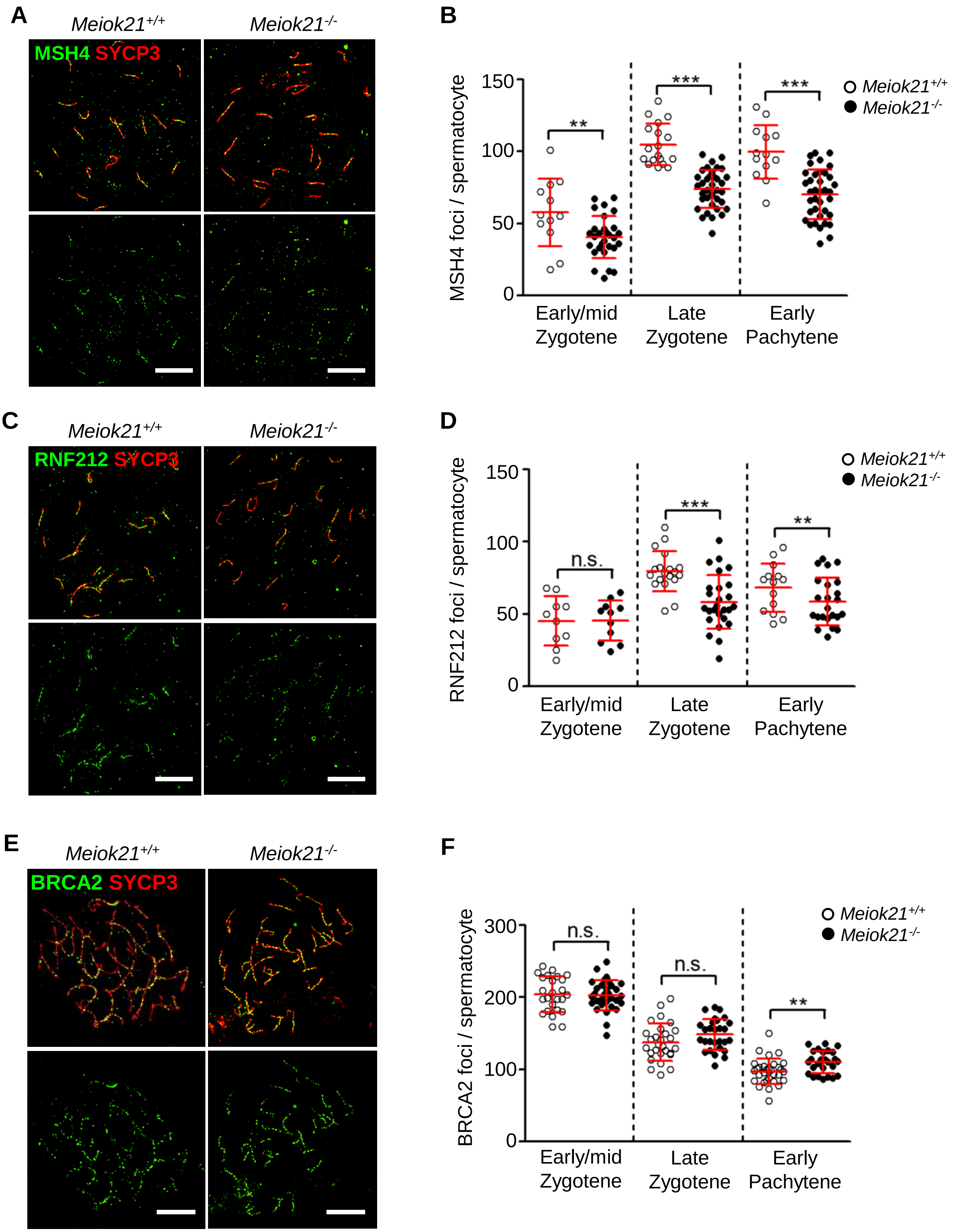

Supplementary Figure S7. Ablation of MEIOK21 decreases MSH4 and RNF212 foci but not BRCA2. The numbers of both MSH4 **(A, B)** and RNF212 **(C, D)** foci were significantly decreased in *Meiok21*$^{-/-}$ spermatocytes compared to WT, but the number of BRCA2 foci **(E, F)** were not altered in *Meiok21*$^{-/-}$ spermatocytes. Scale bar, 10 μm (**A, C, E**). **(B, D, F)** The quantifications of the numbers of MSH4, RNF212 and BRCA2 foci in WT and *Meiok21*$^{-/-}$ spermatocytes. For each panel from left to right, n=12, 29, 18, 36, 13, 37 (**B**); n=10, 11, 19, 26, 15, 23 (**D**); n=25, 31, 28, 28, 31, 26 (**F**). Error bar, mean ± SD (**B, D, F**).
